# Valuing invisible catches: Estimating the global contribution by women to small-scale marine capture fisheries production

**DOI:** 10.1371/journal.pone.0228912

**Published:** 2020-03-04

**Authors:** Sarah Harper, Marina Adshade, Vicky W. Y. Lam, Daniel Pauly, U. Rashid Sumaila

**Affiliations:** 1 Fisheries Economics Research Unit, Institute for the Oceans and Fisheries, The University of British Columbia, Vancouver, British Columbia, Canada; 2 Vancouver School of Economics, The University of British Columbia, Vancouver, British Columbia, Canada; 3 Changing Ocean Research Unit, Institute for the Oceans and Fisheries, The University of British Columbia, Vancouver, British Columbia, Canada; 4 *Sea Around Us*, Institute for the Oceans and Fisheries, The University of British Columbia, Vancouver, British Columbia, Canada; Aristotle University of Thessaloniki, GREECE

## Abstract

The role that women play in fisheries around the world is receiving increasing international attention yet the contributions by women to fisheries catches continues to be overlooked by society, industry and policy makers. Here, we address this lack of visibility with a global estimation of small-scale fisheries catches by women. Our estimates reveal that women participate in small-scale fishing activities in all regions of the world, with approximately 2.1 million (± 86,000) women accounting for roughly 11% (± 4%) of participants in small-scale fishing activities, i.e., catching roughly 2.9 million (± 835,000) tonnes per year of marine fish and invertebrates. The landed value of the catch by women is estimated at USD 5.6 billion (± 1.5 billion), with an economic impact of USD 14.8 billion per year (± 4 billion), which is equivalent to 25.6 billion real 2010 dollars (± 7.2 billion). These catches are mostly taken along the shoreline, on foot, or from small, non-motorized vessels using low-technology, low-emission gears in coastal waters. Catches taken by women are often for home consumption, and thus considered part of the subsistence sub-sector. However, in many contexts, women also sell a portion of their catch, generating income for themselves and their families. These findings underscore the significant role of women as direct producers in small-scale fisheries value chains, making visible contributions by women to food and livelihood security, globally.

## Introduction

“The lack of acknowledgment of women’s fishing participation or of the significant contribution to the livelihoods of coastal people is due, in part, to the non-remuneration of their fishing activities. The lack of data and appropriate economic valuation of subsistence fisheries result in women’s fishing activities not being included in most official statistics.” p.1 [1].

Fishing has long been considered a male domain, i.e., it is often assumed for social, cultural, or religious reasons that women do not participate in fishing activities [2]; however, in the late 1980s women’s fishing activities gained some recognition after colleagues [3,4] wrote about the valuable contributions by women to fisheries economies and marine derived food security around the world. Since then, a growing number of publications and initiatives have highlighted the importance of women to the marine fisheries sector in coastal contexts around the world. Several recent high-level fisheries reports and policy instruments have added to this momentum, emphasizing gender equality as an integral component of efforts to secure coastal livelihoods and the wellbeing of men and women in fishing communities around the world [5–7]. However, despite growing attention to women and gender in fisheries, gender considerations continue to be under-emphasized in fisheries policies and management worldwide [8,9].

Fisheries’ data collection and management efforts often focus on large-scale commercial fisheries, paying much less attention to small-scale fishing activities, especially those for home consumption (i.e., subsistence), and particularly, those where small fish and invertebrates are collected from shore, also known as ‘gleaning’ [10,11]. Since these activities are often not perceived as ‘fishing’, and the people involved may not refer to themselves as ‘fishers/ fishermen/ fisherwomen’, designing fisheries surveys to account for these activities is challenging [12]. This is particularly the case for shellfish fisheries where women have a strong presence in many parts of the world [13–15], and that are notoriously data-poor, with catch records missing or underestimated in national datasets [16]. Recent studies have calculated how productive, despite their invisibility, shellfish fisheries can be in terms of volume of catch relative to other fishing activities, that are dominated by men [8,17,18]. This perception bias, even by trained practitioners, continues today with the collection of shellfish, often by women and children, going unnoticed by fisheries scientists, managers and policy makers, despite the substantial contributions these make to food and livelihood security [10,19]. While there have been increasing efforts to highlight these contributions with the long standing work of the Gender in Aquaculture and Fisheries Section (GAFS) of the Asian Fisheries Society (www.genderaquafish.org), the International Collective in Support of Fishworkers (www.icsf.net/), the Pacific Community (www.spc.int/), the Food and Agriculture Organization of the United Nations (FAO: www.fao.org/fishery/topic/16605/en), WorldFish (www.worldfishcenter.org/), and many other local organizations, gender indicators in fisheries are limited, in many cases only representing industrial processing or other post-harvest employment, where women are more visible and their participation recorded in national labour statistics.

Many qualitative accounts of cultures and contexts around the world describe the participation by women in the collection of marine biomass (i.e., seaweed, fish, and invertebrates), but these activities are often not reflected in fisheries’ statistics and census data or even considered fishing at all. The FAO has made substantial efforts in recent years to improve the state of sex-disaggregated employment statistics for the fisheries and aquaculture sectors, but these efforts are constrained by the voluntary reporting of national data [20]. Currently there is no standardized global dataset containing sex-disaggregated fisheries data that can be used to highlight the contributions by women in fisheries and assess the gendered impacts of policies or changes to fisheries. Sex-disaggregated data, which accounts for men and women separately, does not capture the complexities of gendered practices and relations that exist in the world; however, when used in combination with other indicators or as part of a broader analysis, sex-disaggregated data are critical to understanding gender-based inequalities. In this study, we interrogate the literature, drawing on a wide variety of data sources, and we consult with researchers working in various fisheries contexts around the world to better understand the contributions by women in small-scale fisheries on a global scale, and highlight these contributions in terms of catch volume and associated landed value. While the focus here is on economic indicators, we recognize that the value of catch goes well beyond the monetary value, with much broader societal benefits, that require a much more expansive set of social indicators [21].

Marine capture fisheries provide food, income, and livelihoods to millions of people globally [22–25]. While it is recognized that some of the most vulnerable and marginalized people in society rely the most on fisheries resources, understanding how fisheries (or changes to fisheries) affect these people is limited [22,26]. Several detailed global studies have documented the geographic variation in dependence on small-scale fisheries [27] and in the vulnerability of fisheries economies to climate change [28], identifying countries and regions that are most dependent on fisheries for food and livelihood security and which will be most vulnerable to changes to fisheries. Small-scale fisheries, where women are more likely to participate, generate catches that have been substantially under-estimated in many countries of the world [29–33]. Overlooking these contributions by women adds to the marginalization of small-scale fishers and fisheries, and although this subsector provides food and livelihoods to millions of people globally [34], it receives far less government support or management attention as industrial sectors [35,36]. Therefore, highlighting the marine fisheries catches taken by women in small-scale fisheries will provide more comprehensive accounting in fisheries with a more inclusive set of actors, activities, and subsectors, further emphasizing the contributions from small-scale marine capture fisheries to food and livelihood security in coastal regions around the world. Accounting for these contributions is crucial for advancing policies and programmes that promote the wellbeing of families, children, and future generations and the sustainability of the resources that support them [10]. Building resilience in coastal communities in the face of global economic and environmental change requires acknowledging the role and contributions of all those involved in marine resource related economies, including men, women, youth and elders [37].

As fisheries policies and governance move towards more human-centered approaches that recognize the interdependent nature of social and ecological systems [38], understanding the role of women and gender in these systems is crucial for developing effective policies and programs that strike a balance between the sustainability of fisheries resources and the viability of fishing communities [39]. The collection and reporting of sex-disaggregated statistics is crucial to a comprehensive understanding of resource use patterns and for ecosystem-based approaches to managing fisheries [40]. In cases where men and women target different species, use different gears, and fish in different habitats [10], a gender lens is necessary to understand the implications of various management strategies, to assess the trade-offs, and to improve the outcomes of fisheries management efforts [40]. For example, understanding gendered patterns of resource use is essential for Marine Protected Area planning that is both ecologically beneficial and socially equitable [41].

The rich body of literature on social-ecological systems and resilience has only recently started to engage gender as a critical variable in understanding fisheries as complex, linked human-nature systems. Some researchers have identified the challenges in bringing together gender perspectives and social-ecological systems analysis in fisheries [42], while others point to the value of such an approach for improving management outcomes [40,43], for understanding governance transformations [44], and for increasing adaptation capacity [45]. As interdisciplinary researchers grapple with how to best integrate gender and all its contextual complexities into their analyses, the continued lack of sex-disaggregated data available to managers and policy makers continues to hinder progress towards gender equality in the fisheries sector.

Broad global initiatives such as the Sustainable Development Goals (SDGs) have the potential to influence policies and programs at a national and local level that respond to the broad range of challenges at the human-environment interface, including fisheries [46,47]. SDG 5, to achieve gender equality and to empower all women and girls, and SDG 14, life below water, provide considerable guidance through detailed targets on how to advance each of these goals. However, advancing these goals requires indicators for taking stock, measuring gaps and assessing progress [48]. The collection of sex-disaggregated data for the fisheries sector is critical to the process of developing policies and programs that aim to sustainably and equitably manage our oceans [49].

Here, we focus on one segment of the fish value chain—resource acquisition. There are many other segments (and inputs) along the fish value chain that involve women and where gender inequalities exist that require policy attention. For example, processing of fish and invertebrates into marketable, tradable, and exportable products is often highly labour intensive, with women providing much of the low-cost labour in this post-harvest activity [50–52]. A comprehensive assessment of the contribution by women to fisheries-related economies, must include the entire length of the catch-to-consumption pathway. However, women’s labour contributions in the processing and marketing subsectors have been highlighted to a much greater extent than fisheries production (i.e., catching fish). Therefore, we focus here on the first segment of the fish value chain, as this is an area where perception bias and assumptions about gender roles have resulted in women being largely invisible in this portion of the fisheries value chain.

## Methods

**Fig 1:**
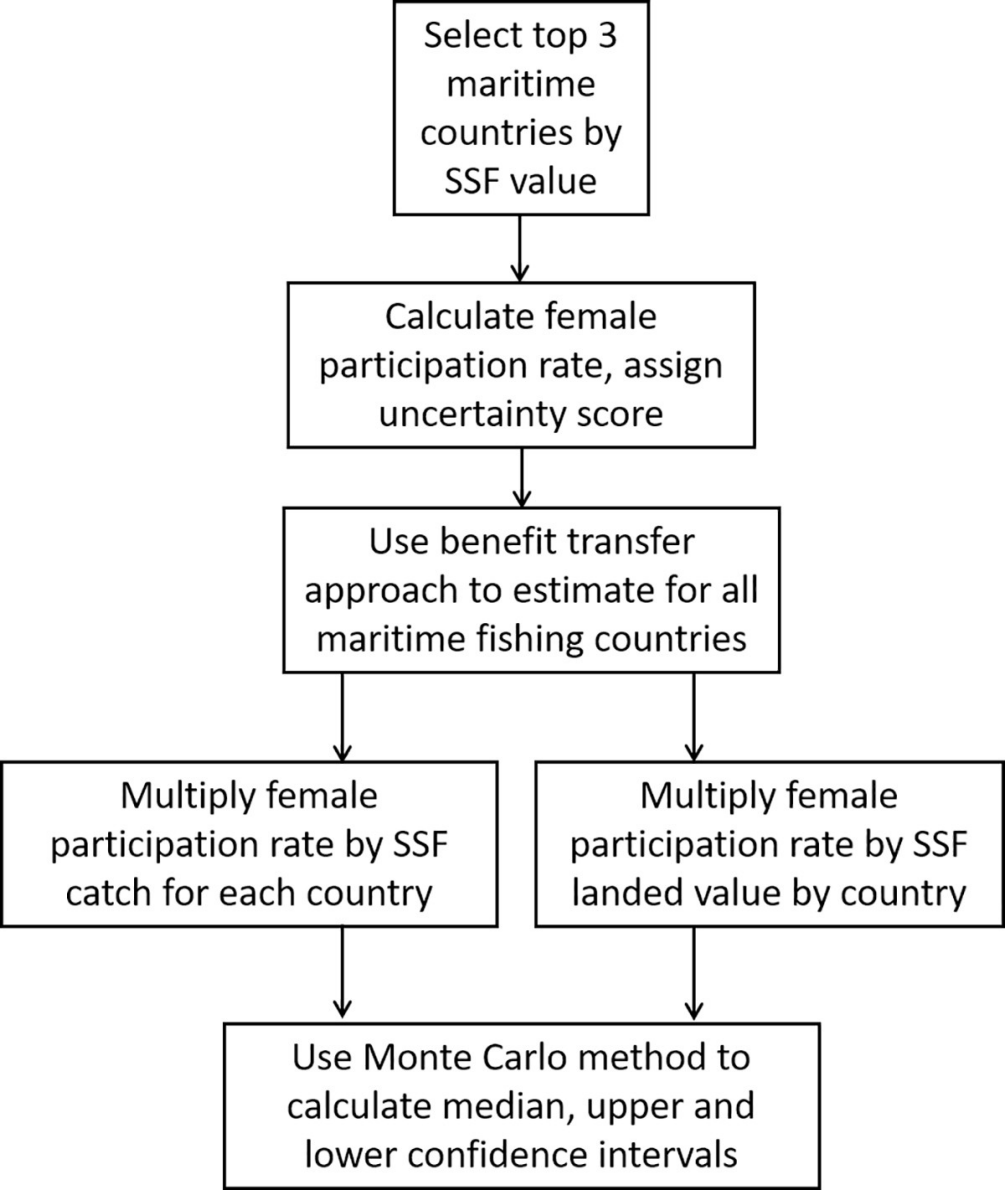
Schematic of stepwise approach for estimating small-scale fisheries catch and landed value by women for all maritime countries of the world.

To estimate the catch and landed value of small-scale fisheries catches by women for all maritime countries of the world, we used a stepwise approach (Fig 1). First, we selected a global subset of countries using national fisheries catch value data provided by the *Sea Around Us* (www.searoundus.org) and the Fisheries Economics Research Unit (http://feru.oceans.ubc.ca/). Dividing the globe into geographic subregions based on the United Nations Statistical Division geographical classification system [53], we selected the top three maritime countries by small-scale fisheries catch value (i.e., landed value of artisanal and subsistence catches; these catches included unreported and unregulated catches but not illegal catches or discards) for each of the 21 subregions of the world, which resulted in a sample size of 62 countries. For Southern Africa and Eastern Asia, we included only 2 maritime countries, while for Eastern Europe and Southeastern Asia we included 4 maritime countries in our sample. Together these 62 countries represent 83% of the global landed value of marine small-scale fisheries catches, thus capturing the majority of the small-scale fishing activity, globally. Our synthesis of data focused on this subset of countries, which were used as the basis for developing indicators for all maritime fishing countries of the world.

Small-scale marine fishing activities, considered here, include fishing, collecting, gleaning and/or harvesting of wild fish and invertebrates (as opposed to farmed, ranched, or aquaculture/mariculture-raised species) from boat or shore, using a range of gear or by hand, for sale (artisanal subsector) or for home consumption (subsistence subsector; [5,32,54]). Recreational fisheries were not included (at least not deliberately). Fishing from boats or operating as a crew onboard a fishing vessel were included under the category of fishing. The definition of small-scale fisheries varies considerably between countries and regions (e.g., [54–56]), so for the purposes of this work, the data correspond to each country’s definition of small-scale fisheries.

### Female participation rates

To estimate participation by women in small-scale fishing activities at the national level, we looked for evidence from existing data sources, either qualitative or quantitative, of women fishing in each of the 62 countries of the global subset. This search for country-specific data included a review of catch reconstruction reports and publications by the *Sea Around Us* (www.seaaroundus.org; [33,57]) project and associated contacts, from primary and/or grey literature sources, and interviews with local experts. For each country, we sought estimates of either female participation rates or number of women participating in small-scale fishing activities. Data sources varied from small-scale fisheries censes to employment statistics, health studies, and socio-economic surveys (See S1 Appendix for country-specific data sources, estimates, and assumptions). Where we calculated the participation rate based on number of female participants, the total small-scale fisheries employment numbers used in our calculations were from colleagues [25], who estimated small-scale fisheries employment, including all small-scale fishing activities, by men and women, even those not captured by national statistics, although not disaggregated by sex. In cases where there were multiple, differing estimates of female participation for a given country, the decision about which source to use was based on the quality of the source (i.e., higher priority was given to peer-reviewed sources), the date of the estimate (i.e., more recent sources were given priority) and extent of coverage (i.e., national level estimates, that included a range of fisheries-related activities and subsectors, were prioritized; see S1 Appendix for description of sources).

Finally, where there was evidence of participation, but quantitative information was unavailable, and to estimate female participation rates for the remaining maritime fishing countries of the world, we used a benefit transfer approach to fill data gaps [58]. This approach involved calculating subregional averages based on data from other countries within that subregion, using direct value transfer to fill data gaps, where the site used to provide the estimate was considered similar to the one lacking data [25,59]. Without a full understanding of the determinants of female participation in fishing across all contexts, our assumption of similarity may not be appropriate in all cases. Within each subregion, we assumed that neighboring and nearby countries likely have similar patterns of female participation in fisheries, because of similar social, cultural, and religious factors that are known to influence female labour force participation rates [60]. However, because socio-cultural factors can vary considerably across short geographic distances, even within subregions, we adjusted some estimates using the rate from another country or subregion based on knowledge of local and regional similarities in social, economic, and colonial history and migration (see S1 Appendix for details). For example, for some overseas territories and island countries where the demographics of the country indicate a dominant ethnic group that is from another geographic subregion, the female participation rate for the subregion was based on similarities in ethnic composition rather than geographic proximity (e.g., Réunion, Cook Islands, Ascension Island, Puerto Rico).

### Catch amount

To calculate catch, the female participation rate in small-scale fishing for each country was multiplied by the total small-scale fisheries catch for that country based on comprehensive catch data from the *Sea Around Us* (www.searoundus.org), that includes reported and unreported catch components. Artisanal and subsistence catches were included in our estimate, as fish and invertebrates caught by women are used both for home consumption and for sale in local markets [6]. Small-scale fisheries catches–including subsistence and artisanal–for the most recent decade (years 2005–2014), were used to calculate the average annual small-scale fisheries catch (in tonnes; S1 Fig). Female participation rates for each country (S2 Fig) were multiplied by the total small-scale fisheries catches for each country to estimate the volume of small-scale fisheries catches by women. This method was tested on several case study countries, where we had independent estimates of catches taken by women (see ‘Validating outputs’ subsection below).

### Landed value and economic impact

The economic value associated with catches taken by women was calculated using female participation rates for small-scale fishing activities and the landed value of small-scale fisheries catches, averaged over a ten-year period, 2005–2014 (S3 Fig). The landed values were derived from *Sea Around Us* catch data [33] and country specific ex-vessel price data from the Fisheries Economics Research Unit [61–63]. We calculated the total revenue (i.e., landed value) of the catch taken by women with subsistence and artisanal catches treated in the same way, assuming that the value of these is similar [62,64]. Given that women often target invertebrates, which can have much higher ex-vessel prices than fish targeted by men, this may underestimate the value of catches by women. For example, in Senegal the ex-vessel price for miscellaneous marine molluscs is USD 2.60 /kg (in 2010), while the ex-vessel price for *Sardinella* spp. is USD 0.58 /kg [63].

Economic impact associated with these catches was estimated using country-specific output multipliers that are based on the direct, indirect, and induced impacts associated with fishing [65]. Landed values and economic impact were presented in 2010 USD, with landed values for each country adjusted to real 2010 dollars using Purchasing Power Parity (PPP) conversion factors presented by the World Bank, which is the number of units of a country's currency required to buy the same amounts of goods and services in the domestic market as US dollars would buy in the United States [66]. Country-specific PPP conversion factors from 2010 were used wherever available. For countries without these data, subregional averages were used.

### Measuring uncertainty

To capture variations in the data used to calculate the amount and value of small-scale fisheries catches attributable to women and the uncertainty around these estimates, we used an approach applied in other data limited contexts, whereby a scoring system is used to calculate confidence intervals around the estimates. This approach is based on the treatment of uncertainty outlined by colleagues [67] for use by the Intergovernmental Panel on Climate Change (IPCC), adapted by adding % values to capture the uncertainty associated with fisheries catches ([33]; Table 1).

**Table 1:** Scoring system for calculating uncertainty associated with estimates of female participation in fisheries, catch amount and value. Adapted from colleagues [67–69].

Score		±%	Corresponding IPCC criteria
4	Very high	10	High agreement & robust evidence
3	High	20	High agreement & medium evidence or medium agreement and robust evidence
2	Low	30	High agreement & limited evidence or medium agreement & medium evidence or low agreement & robust evidence.
1	Very low	50	Less than high agreement and less than robust evidence

Female participation rate estimates were given a score from 1 to 4, based on the quality of the data, evaluated from the ‘agreement’ of sources and the ‘robustness’ of the available evidence. High agreement occurred when there were multiple, independent sources indicating similar estimates, and evidence was considered robust when the source was a peer-reviewed study or a detailed, comprehensive census providing national-level coverage (Table 2). Data taken from the grey literature, based on a regional average or single case-study, scaled-up to a national estimate were considered less robust. Each score is associated with a corresponding percentage (Table 2), which is then used to calculate the confidence intervals associated with catch and value estimates. For example, an estimate with an uncertainty score of 1, which is associated with the highest degree of uncertainty, had a confidence interval range of ± 50%, while a score of 4 has the lowest degree of uncertainty and a confidence interval range of ± 10%. Median values and 95% confidence intervals were calculated using a Monte Carlo simulation method, which has been used previously for fisheries catch and value data where there is considerable uncertainty associated with the data [25]. Here, we used this method to calculate the median catch and 95% confidence interval based on 10,000 iterations of the simulation and assuming a uniform distribution, which is the distribution used in calculating uncertainty associated with similar indicators, for example, in developing the International Union for Conservation of Nature’s Gender and Environment Index [25,70].

**Table 2:** Criteria for assessing the quality of evidence used in estimating the contributions by women in the fisheries sector. Adapted from colleagues [33,67].

**Agreement**	High agreement	> 2 data sources, no conflicting accounts found.
	Medium agreement	> 1 data source, conflicting accounts which could be resolved
	Less than high agreement	≤ 1 data source, conflicting accounts that could not be resolved.
**Robustness**	Robust evidence	data (qualitative and quantitative) from peer-reviewed source or comprehensive census; estimate covers entire country;
	Medium robustness	data from case-study, scaled up to country level; census data not comprehensive (i.e. overlooks labour by women)
	Less than robust	estimate based on regional or sub-regional average.

### Validating outputs

To refine our estimates of female participation in small-scale fishing, we consulted with local experts to verify the results, wherever possible. For each of the 62 countries, emails were sent to individuals with expertise on gender and/or fisheries for a given country. Feedback was received for approximately one third of the countries, which were used to improve estimates and better understand the data and their limitations. For countries where we determined, through this inquiry, that women do not participate at all in small-scale fishing (i.e., a female participation of zero), we consulted with additional experts to verify this information. Validation of catch and value estimates were also done, wherever possible, for countries where published data existed on marine fisheries catches by women, calculated independently from this study, e.g., Senegal [71], Tanzania (S1 Appendix), Samoa (S1 Appendix), and other Pacific Island countries [17].

## Results

Globally, marine small-scale fisheries production activities involve an estimated 2.1 million women (± 86,000), who mainly target invertebrates from intertidal and nearshore habitats, representing approximately 11% of small-scale fishers worldwide (± 4%; S1 Table for a full list of countries with female participation rates and numbers of women). Regionally, female participation rates in fishing activities were estimated to be highest in Oceania, with average female participation of 45% (± 15%) in Melanesia and 27% (± 9%) in Micronesia, while the lowest participation by women in fishing activities was estimated for Western Asia and Eastern Europe (2 ± 1%; Table 3). The overall average participation rate for Asia was estimated here to be 7% (± 2%), with higher rates for Eastern Asia (16 ± 6%) and Southeastern Asia (12 ± 4%) than for Southern Asia (3 ±1%) and Western Asia (2 ± 1%). In Africa, Eastern Africa had the highest participation rate at 26% (± 11%) whereas Northern Africa had the lowest at 2% (± 1%).

**Table 3:** Estimated contributions by women in small-scale fisheries, including participation rates and numbers, catch weight and landed value.

Geographic Area	Female participation rate	Number of female participants	Catch by women^a^ (10^3^ t)	Lower limit (2.5%)	Upper limit (97.5%)	LV in 10^6^ 2010 USD	LV in 10^6^ Real 2010 USD^b^
**Africa**	**0.10**	**237,470**	**262**	**175**	**351**	**450**	**1,081**
Eastern Africa	0.26	104,900	121	82	161	203	563
Middle Africa	0.05	13,500	23	12	34	48	85
Northern Africa	0.02	18,000	4	3	5	9	25
Southern Africa	0.13	5,800	9	7	11	32	52
Western Africa	0.05	95,270	106	71	141	157	356
**Americas**	**0.13**	**912,870**	**776**	**593**	**955**	**1,701**	**2,236**
Caribbean	0.10	305,700	19	12	25	46	67
Central America	0.06	8,480	10	6	14	18	35
Northern America	0.10	24,190	127	92	161	432	429
South America	0.25	574,500	621	482	755	1,205	1,705
**Asia**	**0.07**	**694,460**	**1,743**	**1,220**	**2,266**	**3,015**	**6,051**
Eastern Asia	0.16	127,800	1,039	736	1,340	1,997	3,367
Southeastern Asia	0.12	316,600	548	372	725	839	2,152
Southern Asia	0.03	246,700	136	99	174	136	441
Western Asia	0.02	3,360	20	14	29	43	99
**Europe**	**0.04**	**7,920**	**60**	**40**	**79**	**164**	**172**
Eastern Europe	0.02	1,450	17	9	25	13	25
Northern Europe	0.03	2,280	14	10	17	31	23
Southern Europe	0.07	3,720	25	18	33	108	113
Western Europe	0.03	470	3	2	4	13	11
**Oceania**	**0.25**	**265,320**	**84**	**61**	**106**	**257**	**337**
Austr. & New Zealand	0.13	5,030	19	14	23	85	69
Melanesia	0.45	237,000	46	33	58	127	211
Micronesia	0.27	20,070	12	8	16	23	27
Polynesia	0.19	3,220	7	6	9	22	29
**Global**	**0.11**	**2,118,040**	**2,925**	**2,089**	**3,757**	**5,587**	**9,877**

Notes

^a^Average catches 2005–2014

^b^Values adjusted using Purchasing Power Parity conversion factors from the World Bank to estimate real (2010) dollars.

In terms of small-scale fisheries catches, globally, women catch approximately 2.9 million (± 835,000) tonnes per year of fish and invertebrates. Catches by women were found to be highest in Asia, estimated at over 1.7 million tonnes per year (± 523,000; Table 3).

In Africa catches by women amounted to over 260,000 tonnes annually (± 88,000) and in Oceania they were estimated at over 80,000 tonnes annually (± 23,000; see S2 Table for catch and landed values for all maritime fishing countries).

**Fig 2:**
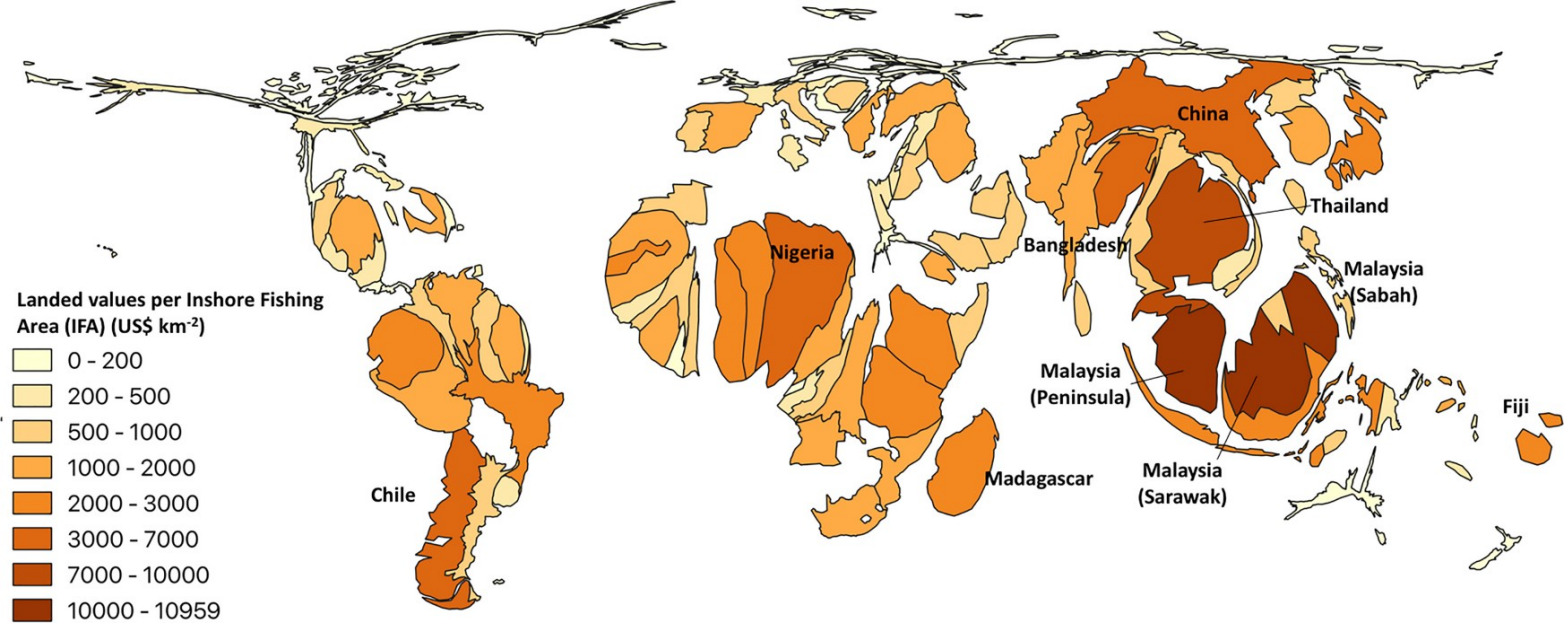
Cartogram of the landed value of catches by women scaled to inshore fishing area for all maritime countries of the world. This figure was created using data obtained with permission from the *Sea Around Us*.

The landed value of catches taken by women were estimated globally at USD 5.6 billion (± 1.5 billion) or 12% of total landed value of small-scale fisheries catches, with an overall economic impact of USD 16.7 billion per year (± 4 billion). When adjusted to real dollars using PPP, the landed value is estimated at over 9.8 billion 2010 dollars (± 2.8 billion; Table 3), with an economic impact of 25.6 billion real dollars (± 7.2 billion). As with catches, the landed value of catches taken by women was highest for Asia, estimated at over USD 3 billion or 6 billion real 2010 dollars when adjusted using PPP (Table 3). Presenting the landed values of small-scale fisheries catches by women scaled to inshore fishing area, highlights the significance of these findings for countries that have a high dependence on small-scale fisheries, such as Asia and African, where SSF catches are especially significant to coastal and rural food and livelihood security (Fig 2).

## Discussion

The contributions by women to small-scale fisheries production, represented here using a currency that is well understood by policymakers (i.e., monetary value), aims to bring new attention to women in fisheries. This research is the first attempt to assemble quantitative estimates of catch by women and associated landed value on a global scale, drawing together existing studies and data, and the knowledge of local experts to highlight and to account for the contributions by women in small-scale fishing activities for all maritime countries of the world. The findings of this study highlight the substantial contributions by women in small-scale fisheries in terms of catch, mostly for subsistence purposes and local markets, and the landed value associated with this catch but also these findings also highlight considerable variation across countries and regions. This variation in participation by women in small-scale fisheries is especially significant if viewed in relation to studies that highlight geographic variation in fisheries dependence and vulnerability [27,28]. In terms of predicting and mitigating the social impacts of climate change, understanding gendered patterns of fishing and highlighting women, as an already vulnerable group in many contexts, are critical to this process. From a public health and wellbeing standpoint, our findings are also significant when considering gendered patterns of household expenditures, where women’s income goes disproportionately towards household provisioning and children’s health and education [8].

The set of indicators developed here, focusing on gender dimensions of small-scale capture fisheries participation, catch, and landed value, complement ongoing efforts led by the FAO, WorldFish and Duke University to highlight the contributions of small-scale fisheries to the Sustainable Development Goals as part of the Illuminating Hidden Harvest project [72]. The FAO has been collecting national fisheries employment statistics from its member countries since the 1950s and has recently started to ask for these data to be disaggregated by sex. While some FAO member countries collect sex-disaggregated fisheries data, many countries lack the capacity to collect these data or resist doing so. Substantial efforts have been made by the FAO to collate sex-disaggregated employment statistics for the fisheries and aquaculture sectors from the member countries who collect and provide them [20]. These efforts are summarized in the 2018 State of World Fisheries and Agriculture report [47]. For the period 2009–2014, approximately 27% of the 194 FAO member countries (n = 52) provided sex-disaggregated fisheries and aquaculture employment data. The State of the World Fisheries Report (2018) indicates that out of this subset of member countries, women represent 14% of all people directly engaged in the fisheries and aquaculture primary sector [47]. Although summary statistics from this dataset have been presented, the data for each country, disaggregated by capture and inland fisheries and aquaculture, were not available for comparison at the time of this study, although our synthesis likely used many similar data sources and came to similar overall results. However, we add to these efforts of participation, estimates of catch and landed value, disaggregated by sex.

While data collected from FAO member countries relies on voluntary reporting, our approach targeted specific countries based on the landed value of small-scale fisheries catches (chosen independently of the availability of data), utilized a range of data sources, including FAO studies and reports. Additionally, we made substantial efforts to validate the data and to address uncertainty in the data by calculating confidence intervals around each estimate. We expanded from our subset of countries to estimate catch by women and associated landed value for all maritime fishing countries of the world using a benefit transfer approach. This approach has been used in other global-scale fisheries studies where data were limited, e.g., fisheries subsidies [73], fisheries employment [25], total catch [74,75], and fishing costs [76] and ex-vessel prices [61–63].

The results of this study build on previous and ongoing efforts at various scales, that recognize the need for a comprehensive set of sex-disaggregated data as required for gender analyses to develop policies that are in line with SDG targets and SSF guidelines on gender equality. However, overcoming the many existing data deficiencies in small-scale fisheries, and especially when it comes to gender indicators and disaggregated data, requires a coordinated effort with the resources and funding needed to collect data in a rigorous and standardized way. The fisheries catch estimates presented here are considered conservative and the uncertainty associated with our estimates is substantial, in part, because of our reliance on secondary data sources. Reducing uncertainty would require the collection of primary data, standardized in terms of when and how the data are collected, across all countries. While recognizing the limitations of the dataset created here, we present these results specifically to invite feedback and criticism from countries, with any misrepresentations as motivation for national fisheries and statistics agencies to start (routinely) collecting sex-disaggregated data as a critical input for improving fisheries management and policies [40].

Our method for estimating catch assumed that men and women fish in the same way, which we know is not always the case. The very limited data available comparing catch per unit effort (CPUE) between men and women [10] indicate that in some fisheries and in certain contexts, women have a higher CPUE than men [10,77], while in other examples, men have a higher CPUE than women when targeting the same species [78,79]. Given the limited sex-disaggregated data on fishing effort or the frequency and duration of fishing activities in relation to catch volume, we assumed these to be constant among men and women. We justify this assumption based on anecdotal evidence that describes men who go fishing, far from shore, for long periods of time and bring back a limited catch, while women collect shellfish, near to shore, for a couple hours per day, amounting to relatively larger catches. In other contexts, men might catch much more than women, based on the types of gear they use. Given the considerable lack of sex-disaggregated data on effort, and/or on the types of fishing activities, species targeted, and gears used by men and women, female participation rates were used as the best available metric, comparable across, and inclusive of all maritime fishing countries of the world. The limitation of using unstandardized participation rates for calculating catch is that, in some cases, this may overestimate catch, while in other cases, this may underestimate it. For this reason, individual country estimates should be interpreted and employed cautiously. For example, where participation by women in small-scale fishing activities is zero, these estimates should be viewed as highly uncertain, as we know that fishing by women in many contexts can be easily overlooked.

While catches attributed to fishing by women represents approximately 5% of the overall landed value of marine fisheries globally (including all small- and large-scale sectors), the contribution to food and livelihood security at local and national levels is non-trivial and must be considered alongside this economic valuation [52]. Additional metrics are urgently needed to fully capture the significance of these contributions in terms of food, nutrition, poverty alleviation, and beyond. This study identifies many gaps that exist when it comes to sex-disaggregated data in fisheries. Information, such as gender-specific target species, habitats fished, gear used and effort, is critical for understanding possible gendered impacts of climate change and developing appropriate mitigation strategies, for Marine Protected Area planning and for understanding fisheries-related food security [41,45,80]. This information is especially critical at a time where there is increasing pressure to align these marine policy dimensions with global targets and strategies to reduce hunger, to alleviate poverty and to promote gender equality [81].

Broadly, this research contributes towards a more complete understanding of fisheries economies and fisheries as social-ecological systems. However, these findings are limited in scope and include substantial uncertainty. As gender roles and relationships are continuously being negotiated, these estimates will change and should be revised accordingly. The dynamic and interconnected social, cultural, economic and ecological factors that shape fisheries systems will also influence these numbers over time. While individual country-level estimates should be used cautiously, we are confident that the findings presented here will contribute to important conversations at local, national, and international levels about how women are seen and valued in the fisheries sector and beyond.

## Supporting information

S1 Fig Map of small-scale fisheries catches averaged over the 2005–2014 time period for all maritime countries of the world. This figure was created using data obtained with permission from the *Sea Around Us*.
(TIF)

S2 Fig Map of female participation rates in small-scale fishing activities for all maritime countries of the world. (TIF)

S3 Fig Map of total landed value of small-scale fisheries catches averaged over the 2005–2014 time period for all maritime countries of the world. This figure was created using data obtained with permission from the *Sea Around Us*.
(TIF)

S1 Table Participation by women in small-scale fishing activities as a participation rate and numbers estimated based on these rates and total number of small-scale fishers. (DOCX)

S2 Table Estimated small-scale marine capture fisheries catch and landed value (in 2010 USD) by women for all maritime countries of the world, including 95% confidence intervals, calculated using a Monte Carlo simulation. (DOCX)

S1 Appendix Country estimates, assumptions and uncertainty scores. (DOC)
