# Supplementary figures and images for: Valuing invisible catches: Estimating the global contribution by women to small-scale marine capture fisheries production
Source: PLoS One. 2020 Mar 4;15(3):e0228912. doi: 10.1371/journal.pone.0228912 (PMC7055739; doi:10.1371/journal.pone.0228912)

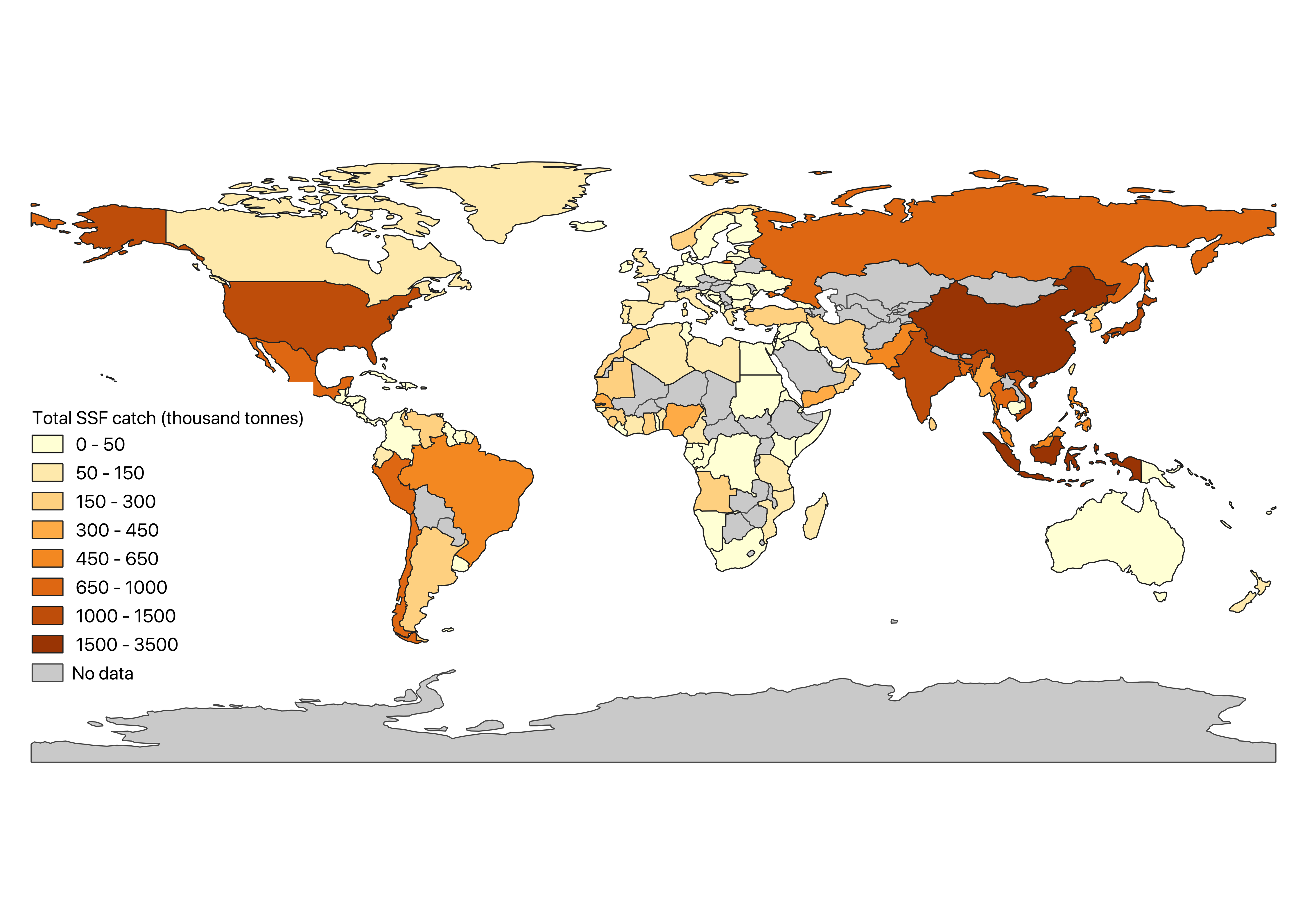

Supplement: S1 Fig — This figure was created using data obtained with permission from the Sea Around Us. (TIF) [file pone.0228912.s001.tif]

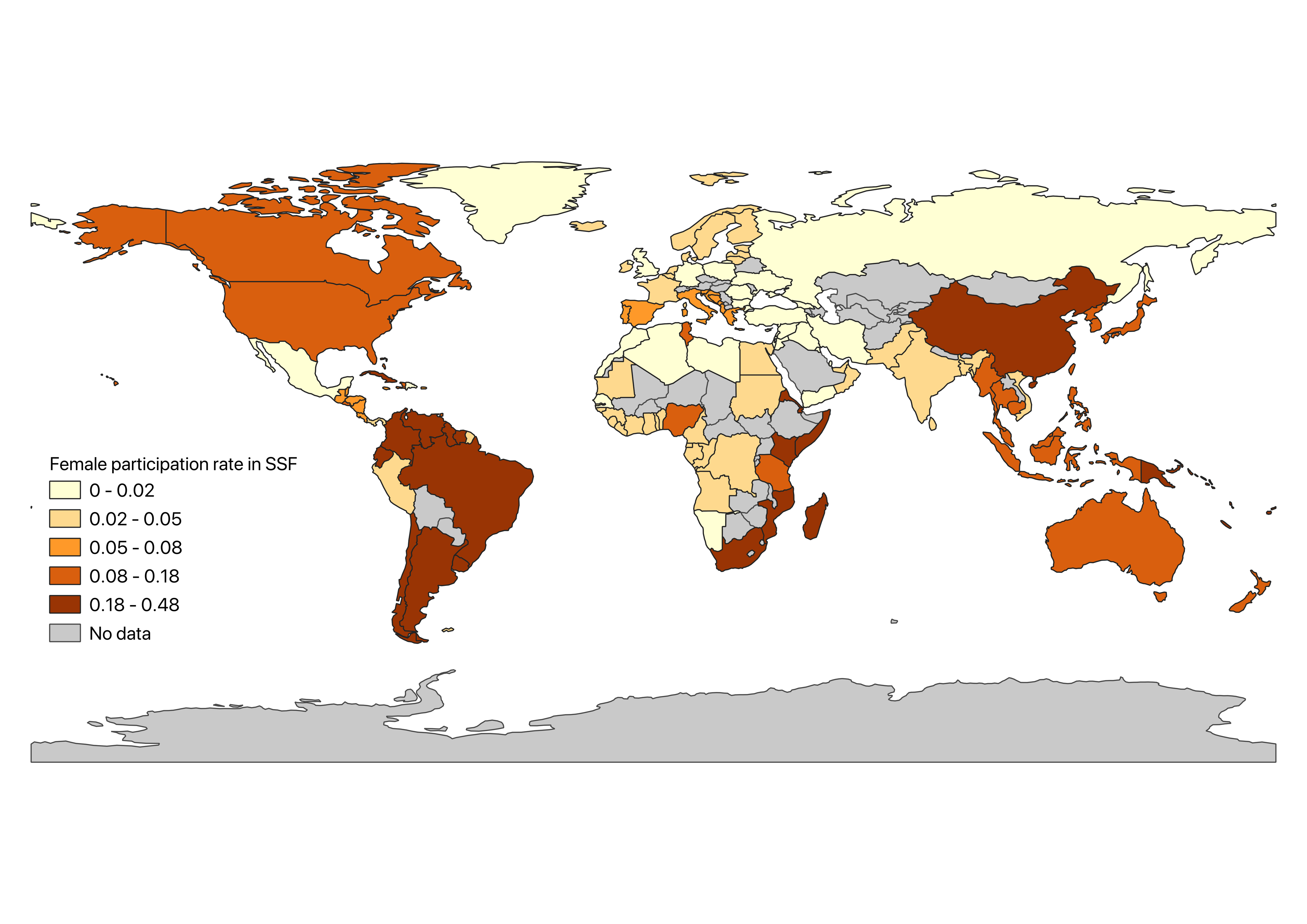

Supplement: S2 Fig — (TIF) [file pone.0228912.s002.tif]

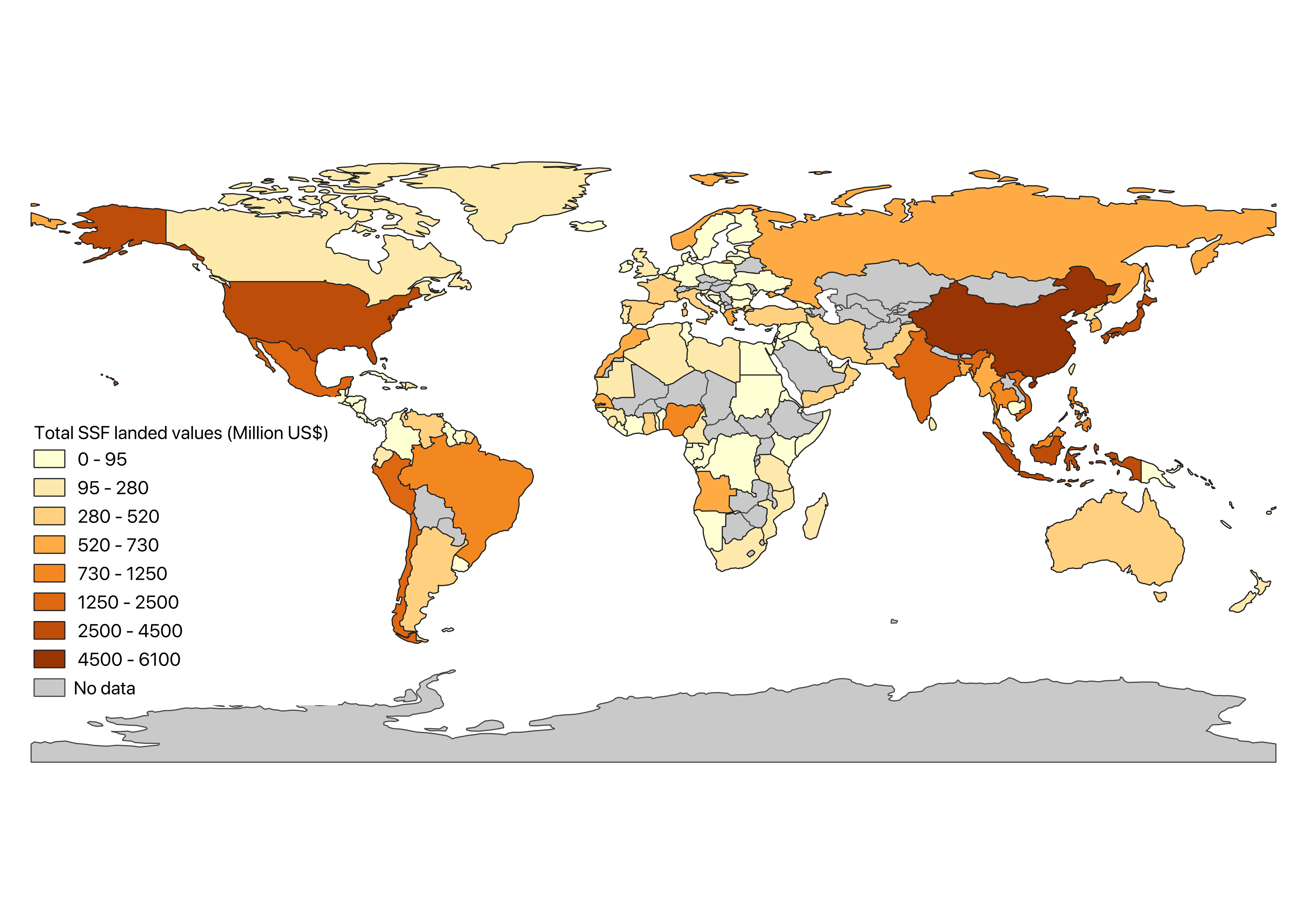

Supplement: S3 Fig — This figure was created using data obtained with permission from the Sea Around Us. (TIF) [file pone.0228912.s003.tif]
