# Supplementary material for: Valuing invisible catches: Estimating the global contribution by women to small-scale marine capture fisheries production
Source: PLoS One. 2020 Mar 4;15(3):e0228912. doi: 10.1371/journal.pone.0228912 (PMC7055739; doi:10.1371/journal.pone.0228912)
Supplement: S1 Table — (DOCX) [file pone.0228912.s004.docx]

| **Geographic Area** | **Female participation rate SSF** | **# of female participants** |
| --- | --- | --- |
| **Africa** | **0.11** | **237,490** |
| **Eastern Africa** | **0.28** | **104,900** |
| Comoros Isl. | 0.28 | 7,991 |
| Djibouti | 0.28 | 193 |
| Eritrea | 0.28 | 1,075 |
| Kenya | 0.28 | 744 |
| Madagascar | 0.46 | 69,000 |
| Mauritius | 0.28 | 1,791 |
| Mozambique | 0.28 | 12,600 |
| Seychelles | 0.28 | 165 |
| Somalia | 0.28 | 6,889 |
| Tanzania | 0.09 | 4,455 |
| **Middle Africa** | **0.05** | **13,500** |
| Angola | 0.05 | 900 |
| Cameroon | 0.05 | 1,500 |
| Congo (ex-Zaire) | 0.05 | 255 |
| Congo, R. of | 0.05 | 10,000 |
| Eq Guinea | 0.05 | 380 |
| Gabon | 0.05 | 125 |
| Sao Tome Prn | 0.05 | 335 |
| **Northern Africa** | **0.03** | **18,000** |
| Algeria | 0.00 | 0 |
| Egypt | 0.03 | 16,750 |
| Libya | 0.00 | 0 |
| Morocco | 0.00 | 0 |
| Sudan | 0.03 | 180 |
| Tunisia | 0.10 | 1,100 |
| **Southern Africa** | **0.10** | **5,800** |
| Namibia | 0.00 | 0 |
| South Africa | 0.20 | 5,800 |
| **Western Africa** | **0.05** | **95,270** |
| Benin | 0.05 | 1,661 |
| Cape Verde | 0.05 | 805 |
| Côte d'Ivoire | 0.05 | 367 |
| Gambia | 0.05 | 1,560 |
| Ghana | 0.00 | 0 |
| Guinea | 0.05 | 4,127 |
| Guinea Bissau | 0.05 | 4,178 |
| Liberia | 0.05 | 856 |
| Mauritania | 0.05 | 1,460 |
| Nigeria | 0.13 | 76,800 |
| Senegal | 0.02 | 1,337 |
| Sierra Leone | 0.05 | 1,560 |
| Togo | 0.05 | 554 |
| **Americas** | **0.14** | **912,870** |
| **Caribbean** | **0.10** | **305,700** |
| Antigua & Barbuda | 0.10 | 600 |
| Bahamas | 0.10 | 2,794 |
| Barbados | 0.10 | 2,483 |
| Cuba | 0.25 | 250,000 |
| Dominica | 0.10 | 228 |
| Dominican Republic | 0.00 | 140 |
| Grenada | 0.10 | 414 |
| Haiti | 0.10 | 31,040 |
| Jamaica | 0.06 | 5,400 |
| Saint Kitts and Nevis | 0.10 | 352 |
| St Vincent | 0.10 | 248 |
| St. Lucia | 0.10 | 662 |
| Trinidad and Tobago | 0.10 | 11,381 |
| **Central America** | **0.06** | **8,480** |
| Belize | 0.06 | 602 |
| Costa Rica | 0.06 | 514 |
| El Salvador | 0.15 | 3,040 |
| Guatemala | 0.06 | 19 |
| Honduras | 0.06 | 2,384 |
| Mexico | 0.00 | 440 |
| Nicaragua | 0.06 | 1,004 |
| Panama | 0.03 | 476 |
| **Northern America** | **0.11** | **24,190** |
| Canada | 0.14 | 2,565 |
| USA | 0.09 | 21,620 |
| **South America** | **0.24** | **574,500** |
| Argentina | 0.24 | 2,178 |
| Brazil | 0.45 | 495,000 |
| Chile | 0.23 | 13,110 |
| Colombia | 0.24 | 24,200 |
| Ecuador | 0.24 | 11,616 |
| Guyana | 0.24 | 11,616 |
| Peru | 0.05 | 2,024 |
| Suriname | 0.24 | 8,954 |
| Uruguay | 0.24 | 266 |
| Venezuela | 0.24 | 5,566 |
| **Asia** | **0.07** | **694,300** |
| **Eastern Asia** | **0.18** | **127,800** |
| China | 0.22 | 68,200 |
| Hong Kong | 0.18 | 1,418 |
| Japan | 0.13 | 31,200 |
| Korea South | 0.18 | 14,175 |
| Taiwan | 0.18 | 12,775 |
| **Southeastern Asia** | **0.11** | **316,600** |
| Brunei | 0.10 | 49 |
| Cambodia | 0.10 | 15,573 |
| Indonesia | 0.10 | 110,000 |
| Malaysia | 0.18 | 17,150 |
| Myanmar | 0.10 | 41,853 |
| Philippines | 0.10 | 28,227 |
| Singapore | 0.10 | 749 |
| Thailand | 0.15 | 34,960 |
| Vietnam | 0.04 | 68,000 |
| **Southern Asia** | **0.03** | **246,700** |
| Bangladesh | 0.05 | 130,000 |
| India | 0.05 | 69,000 |
| Iran | 0.00 | 0 |
| Maldives | 0.03 | 480 |
| Pakistan | 0.03 | 44,800 |
| Sri Lanka | 0.03 | 2,336 |
| **Western Asia** | **0.02** | **3,360** |
| Bahrain | 0.02 | 950 |
| Cyprus | 0.02 | 15 |
| Georgia | 0.02 | 30 |
| Israel | 0.02 | 22 |
| Jordan | 0.02 | 2 |
| Kuwait | 0.02 | 467 |
| Lebanon | 0.02 | 233 |
| Oman | 0.03 | 93 |
| Qatar | 0.02 | 40 |
| Saudi Arabia | 0.02 | 183 |
| Syria | 0.02 | 10 |
| Turkey | 0.02 | 480 |
| United Arab Emirates | 0.03 | 71 |
| Yemen | 0.02 | 767 |
| **Europe** | **0.04** | **7,920** |
| **Eastern Europe** | **0.02** | **1,450** |
| Bulgaria | 0.02 | 4 |
| Poland | 0.02 | 90 |
| Romania | 0.02 | 460 |
| Russian Federation | 0.02 | 400 |
| Ukraine | 0.02 | 500 |
| **Northern Europe** | **0.03** | **2,280** |
| Denmark | 0.04 | 263 |
| Estonia | 0.03 | 149 |
| Finland | 0.03 | 1,181 |
| Iceland | 0.03 | 116 |
| Ireland | 0.03 | 51 |
| Latvia | 0.03 | 59 |
| Lithuania | 0.03 | 67 |
| Norway | 0.03 | 93 |
| Sweden | 0.03 | 59 |
| United Kingdom | 0.02 | 240 |
| **Southern Europe** | **0.08** | **3,720** |
| Albania | 0.08 | 276 |
| Croatia | 0.08 | 123 |
| Greece | 0.07 | 321 |
| Italy | 0.08 | 2,160 |
| Malta | 0.08 | 15 |
| Portugal | 0.08 | 192 |
| Spain | 0.09 | 630 |
| **Western Europe** | **0.03** | **470** |
| Belgium | 0.03 | 17 |
| France | 0.03 | 300 |
| Germany | 0.01 | 20 |
| Netherlands | 0.05 | 135 |
| **Oceania** | **0.31** | **265,400** |
| **Australia and New Zealand** | **0.13** | **5,030** |
| Australia | 0.13 | 4,480 |
| New Zealand | 0.13 | 550 |
| **Melanesia** | **0.45** | **237,000** |
| Fiji | 0.46 | 17,020 |
| Papua New Guinea | 0.48 | 216,000 |
| Solomon Islands | 0.42 | 3,234 |
| Vanuatu | 0.45 | 861 |
| **Micronesia** | **0.31** | **20,070** |
| Kiribati | 0.35 | 8,050 |
| Marshall Islands | 0.31 | 1,595 |
| Micronesia | 0.25 | 9,500 |
| Nauru | 0.31 | 123 |
| Palau | 0.32 | 800 |
| **Polynesia** | **0.19** | **3,220** |
| Samoa | 0.20 | 2,400 |
| Tonga | 0.17 | 816 |
| **Globally** | **0.11** | **2,118,040** |
