# Supplementary material for: Valuing invisible catches: Estimating the global contribution by women to small-scale marine capture fisheries production
Source: PLoS One. 2020 Mar 4;15(3):e0228912. doi: 10.1371/journal.pone.0228912 (PMC7055739; doi:10.1371/journal.pone.0228912)
Supplement: S2 Table — (DOCX) [file pone.0228912.s005.docx]

| **Geographic Area** | **Catch by women** | **95% Confidence Interval, Catch ±** | **Landed Value (LV) of catch by Women (2010 USD)** | **95% Confidence Interval, LV**  **(2010 USD) ±** |
| --- | --- | --- | --- | --- |
| **Africa** | 262,000 | 88,000 | 450,000,000 | 142,000,000 |
| **Eastern Africa** | 121,000 | 39,500 | 203,000,000 | 67,000,000 |
| Comoros | 5,140 | 2,455 | 8,031,000 | 3,800,000 |
| Djibouti | 564 | 267 | 1,255,000 | 602,900 |
| Eritrea | 1,100 | 516 | 1,551,000 | 740,400 |
| Kenya | 3,580 | 1,715 | 9,277,000 | 4,403,000 |
| Madagascar | 52,300 | 15,000 | 98,040,000 | 27,770,000 |
| Mauritius | 1,350 | 641 | 2,863,000 | 1,360,000 |
| Mayotte | 660 | 313 | 3,175,000 | 1,503,000 |
| Mozambique | 31,500 | 9,000 | 39,420,000 | 11,218,000 |
| Réunion | 125 | 60 | 593,100 | 282,000 |
| Seychelles | 1,510 | 714 | 2,186,000 | 1,040,000 |
| Somalia | 13,200 | 6,262 | 18,980,000 | 9,039,000 |
| Tanzania | 10,000 | 2,870 | 17,310,000 | 4,920,000 |
| **Middle Africa** | 23,000 | 11,000 | 48,000,000 | 23,040,000 |
| Angola | 11,500 | 5,430 | 29,820,000 | 14,320,000 |
| Cameroon | 6,280 | 2,960 | 6,961,000 | 3,328,000 |
| Congo  (ex-Zaire) | 638 | 304 | 1,699,000 | 806,700 |
| Congo, R. of | 1,750 | 835 | 3,709,000 | 1,761,000 |
| Equatorial Guinea | 447 | 213 | 1,208,000 | 574,300 |
| Gabon | 2,000 | 956 | 3,907,000 | 1,858,000 |
| Sao Tome & Principe | 447 | 213 | 831,000 | 393,900 |
| **Northern Africa** | 4,000 | 1,000 | 9,000,000 | 2,300,000 |
| Algeria | 0 | 0 | 0 | 0 |
| Egypt | 956 | 454 | 1,878,000 | 885,700 |
| Libya | 0 | 0 | 0 | 0 |
| Morocco | 0 | 0 | 0 | 0 |
| Sudan | 52 | 25 | 141,400 | 66,980 |
| Tunisia | 2,880 | 548 | 7,250,000 | 1,378,000 |
| **Southern Africa** | 9,000 | 1,700 | 32,000,000 | 6,150,000 |
| Namibia | 0 | 0 | 0 | 0 |
| South Africa | 9,020 | 1,720 | 32,360,000 | 6,148,000 |
| **Western Africa** | 106,000 | 35,000 | 157,000,000 | 43,500,000 |
| Ascension Isl. | 1 | <0.5 | 2,544 | 1,228 |
| Benin | 3,400 | 1,606 | 3,765,000 | 1,779,000 |
| Cape Verde | 400 | 192 | 1,059,000 | 500,300 |
| Cote D'Ivoire | 2,600 | 1,240 | 3,500,000 | 1,652,000 |
| Gambia | 4,130 | 1,970 | 3,122,000 | 1,481,000 |
| Ghana | 6,190 | 2,958 | 7,002,000 | 3,322,000 |
| Guinea | 8,560 | 4,023 | 6,412,000 | 3,067,000 |
| Guinea-Bissau | 1,750 | 832 | 2,721,000 | 1,303,000 |
| Liberia | 1,290 | 614 | 1,592,000 | 760,200 |
| Mauritania | 10,100 | 4,770 | 9,044,000 | 4,270,000 |
| Nigeria | 43,900 | 8,370 | 93,410,000 | 17,750,000 |
| Senegal | 9,080 | 1,730 | 16,000,000 | 3,050,000 |
| Sierra Leone | 13,000 | 6,190 | 8,185,000 | 3,928,000 |
| Togo | 1,050 | 500 | 1,240,000 | 588,700 |
| Tristan da Cunha Isl. | 1 | 1 | 2,097 | 990 |
| **Americas** | 776,000 | 181,000 | 1,701,000,000 | 406,500,000 |
| **Caribbean** | 19,000 | 6,600 | 46,000,000 | 16,800,000 |
| Anguilla | 164 | 78 | 1,063,000 | 502,800 |
| Antigua Barb | 390 | 185 | 1,258,000 | 597,300 |
| Aruba | 20 | 9 | 92,880 | 44,330 |
| Bahamas | 494 | 235 | 1,923,000 | 911,400 |
| Barbados | 266 | 127 | 322,500 | 154,500 |
| Bermuda | 61 | 29 | 405,500 | 193,600 |
| Bonaire | 30 | 14 | 108,000 | 50,930 |
| British Virgin Isl. | 388 | 184 | 1,960,000 | 932,100 |
| Cayman Isl. | 0.20 | 0.10 | 1,417 | 677 |
| Cuba | 6,670 | 1,271 | 15,920,000 | 3,026,000 |
| Curacao | 125 | 59 | 505,300 | 240,100 |
| Dominica | 159 | 75 | 432,000 | 207,600 |
| Dominican Republic | 20 | 6 | 38,370 | 11,010 |
| Grenada | 242 | 115 | 469,800 | 222,500 |
| Guadeloupe | 1,100 | 516 | 2,432,000 | 1,149,000 |
| Haiti | 2,510 | 1,185 | 4,167,400 | 1,987,000 |
| Jamaica | 2,070 | 591 | 3,578,000 | 1,021,000 |
| Martinique | 703 | 339 | 2,025,000 | 967,100 |
| Montserrat | 6 | 3 | 20,280 | 9,633 |
| Puerto Rico | 78 | 37 | 269,100 | 128,500 |
| Saba & Saint Eustaius | 32 | 15 | 298,300 | 141,600 |
| Saint Kitts & Nevis | 170 | 81 | 717,300 | 341,500 |
| Saint Lucia | 141 | 67 | 274,300 | 129,700 |
| Saint Vincent & the Grenadines | 164 | 78 | 693,700 | 327,400 |
| Sint Maarten | 7 | 3 | 27,460 | 13,070 |
| St Barthelemy | 36 | 17 | 201,000 | 95,350 |
| St Martin | 80 | 38 | 460,500 | 216,900 |
| Trinidad & Tobago | 1,330 | 633 | 3,024,000 | 1,426,000 |
| Turks & Caicos Isl. | 1,140 | 541 | 3,287,000 | 1,543,000 |
| US Virgin Isl. | 121 | 57 | 437,100 | 209,500 |
| **Central America** | 10,000 | 3,700 | 18,000,000 | 6,600,000 |
| Belize | 398 | 189 | 676,200 | 323,300 |
| Costa Rica | 557 | 265 | 895,100 | 425,600 |
| El Salvador | 2,510 | 711 | 2,326,000 | 660,800 |
| Guatemala | 1,670 | 791 | 2,118,000 | 1,008,000 |
| Honduras | 834 | 395 | 2,331,000 | 1,100,000 |
| Mexico | 2,010 | 575 | 4,065,000 | 1,160,000 |
| Nicaragua | 1,120 | 533 | 2,521,000 | 1,208,000 |
| Panama | 883 | 252 | 2,653,000 | 755,400 |
| **Northern America** | 127,000 | 34,400 | 432,000,000 | 119,000,000 |
| Canada | 8,600 | 817 | 20,150,000 | 1,913,000 |
| Greenland | 244 | 46 | 763,700 | 144,800 |
| USA | 117,700 | 23,500 | 411,200,000 | 117,000,000 |
| **South America** | 621,000 | 136,400 | 1,205,000,000 | 264,000,000 |
| Argentina | 46,300 | 22,080 | 69,010,000 | 32,690,000 |
| Brazil | 204,000 | 38,670 | 488,100,000 | 92,580,000 |
| Chile | 227,000 | 21,600 | 371,800,000 | 35,380,000 |
| Colombia | 3,650 | 1,730 | 7,104,000 | 3,375,000 |
| Ecuador | 25,600 | 12,150 | 37,580,000 | 17,750,000 |
| French Guiana | 875 | 416 | 2,217,000 | 1,051,000 |
| Guyana | 7,940 | 3,735 | 11,760,000 | 5,546,000 |
| Peru | 36,300 | 3,430 | 73,140,000 | 6,950,000 |
| Suriname | 9,200 | 4,350 | 13,820,000 | 6,582,000 |
| Uruguay | 7,440 | 3,539 | 9,433,000 | 4,497,00 |
| Venezuela | 52,500 | 24,695 | 120,630,000 | 57,580,000 |
| **Asia** | 1,743,000 | 523,000 | 3,015,000,000 | 872,600,000 |
| **Eastern Asia** | 1,039,000 | 301,600 | 1,997,000,000 | 552,600,000 |
| China | 746,000 | 211,600 | 1,342,000,000 | 381,800,000 |
| Hong Kong | 643 | 306 | 1,083,000 | 514,800 |
| Japan | 173,000 | 33,000 | 487,000,000 | 92,260,000 |
| Korea (North) | 30,800 | 14,700 | 26,660,000 | 12,660,000 |
| Korea (South) | 74,700 | 35,400 | 119,500,000 | 56,000,000 |
| Taiwan | 13,700 | 6,540 | 19,970,000 | 9,457,000 |
| **Southeastern Asia** | 548,000 | 176,600 | 839,000,000 | 267,800,000 |
| Brunei | 1,230 | 580 | 2,053,000 | 967,300 |
| Cambodia | 2,020 | 964 | 2,714,000 | 1,288,000 |
| Indonesia | 169,000 | 48,160 | 253,400,000 | 72,150,000 |
| Malaysia | 113,000 | 32,180 | 213,100,000 | 60,620,000 |
| Myanmar | 40,800 | 19,470 | 50,600,000 | 24,050,000 |
| Philippines | 62,110 | 29,500 | 95,310,000 | 45,350,000 |
| Singapore | 185 | 87 | 335,000 | 159,000 |
| Thailand | 114,000 | 32,400 | 170,300,000 | 48,480,000 |
| Timor Leste | 591 | 282 | 1,037,000 | 496,900 |
| Vietnam | 45,300 | 12,980 | 50,180,000 | 14,280,000 |
| **Southern Asia** | 136,000 | 37,600 | 136,000,000 | 36,800,000 |
| Bangladesh | 41,600 | 11,830 | 32,750,000 | 9,342,000 |
| India | 67,300 | 12,790 | 75,710,000 | 14,310,000 |
| Iran | 0 | 0 | 0 | 0 |
| Maldives | 2,006 | 949 | 4,560,000 | 2,165,000 |
| Pakistan | 17,500 | 8,270 | 16,180,000 | 7,611,000 |
| Sri Lanka | 7,970 | 3,780 | 7,222,000 | 3,408,000 |
| **Western Asia** | 20,300 | 7,270 | 43,300,000 | 15,290,000 |
| Bahrain | 906 | 425 | 3,369,000 | 1,610,000 |
| Gaza Strip | 40 | 19 | 96,350 | 45,900 |
| Georgia | 91 | 43 | 96,920 | 45,900 |
| Iraq | 112 | 53 | 305,600 | 145,300 |
| Israel | 18 | 9 | 62,730 | 29,580 |
| Jordan | 4 | 2 | 7,593 | 3,635 |
| Kuwait | 284 | 135 | 510,700 | 244,000 |
| Lebanon | 125 | 59 | 231,500 | 110,300 |
| North Cyprus | 10 | 5 | 61,150 | 29,180 |
| Oman | 5,800 | 1,100 | 12,430,000 | 2,354,000 |
| Qatar | 587 | 279 | 2,643,000 | 1,260,000 |
| Saudi Arabia | 0 | 0 | 0 | 0 |
| South Cyprus | 30 | 14 | 90,950 | 43,340 |
| Syria | 64 | 31 | 117,400 | 55,700 |
| Turkey | 3,830 | 1,090 | 9,473,000 | 2,706,000 |
| United Arab Emirates | 2,130 | 1,010 | 7,307,000 | 3,464,000 |
| Yemen | 6,290 | 3,002 | 6,575,000 | 3,144,000 |
| **Europe** | 60,000 | 19,900 | 164,000,000 | 43,000,000 |
| **Eastern Europe** | 17,000 | 8,100 | 13,000,000 | 6,000,000 |
| Bulgaria | 15 | 7 | 38,430 | 18,270 |
| Poland | 361 | 103 | 238,500 | 67,980 |
| Romania | 20 | 9 | 44,110 | 20,990 |
| Russian Federation | 16,800 | 7,960 | 12,230,000 | 5,849,000 |
| Ukraine | 221 | 63 | 200,600 | 57,060 |
| **Northern Europe** | 14,000 | 3,400 | 31,000,000 | 7,400,000 |
| Channel Isl. | 15 | 7 | 46,320 | 21,780 |
| Denmark | 1,320 | 251 | 2,641,000 | 502,000 |
| Estonia | 181 | 85 | 61,120 | 28,800 |
| Faeroe Isl. | 468 | 221 | 1,050,000 | 501,500 |
| Falkland Isl. | 1 | 1 | 2,288 | 1,082 |
| Finland | 283 | 136 | 120,300 | 56,910 |
| Iceland | 768 | 364 | 1,544,000 | 737,000 |
| Ireland | 277 | 131 | 957,100 | 452,800 |
| Latvia | 194 | 92 | 78,380 | 37,250 |
| Lithuania | 28 | 13 | 18,850 | 8,985 |
| Norway | 8,380 | 1,600 | 21,130,000 | 4,020,000 |
| Saint Helena | 4 | 2 | 12,870 | 6,093 |
| Sweden | 377 | 180 | 304,100 | 144,100 |
| UK | 1,230 | 349 | 3,225,000 | 913,900 |
| **Southern Europe** | 25,000 | 7,700 | 108,000,000 | 27,200,000 |
| Albania | 31 | 15 | 32,140 | 15,230 |
| Azores Isl. | 93 | 45 | 154,000 | 73,150 |
| Bosnia | 4 | 2 | 5,844 | 2,783 |
| Croatia | 951 | 451 | 1,308,000 | 621,600 |
| Greece | 6,600 | 1,343 | 36,570,000 | 7,558,000 |
| Italy | 4,390 | 925 | 31,040,000 | 6,553,000 |
| Madeira Isl. | 189 | 90 | 648,900 | 308,700 |
| Malta | 61 | 29 | 241,300 | 114,300 |
| Montenegro | 34 | 16 | 50,900 | 24,300 |
| Portugal | 6,260 | 2,300 | 12,920,000 | 6,175,000 |
| Slovenia | 13 | 6 | 60,754 | 28,620 |
| Spain | 6,780 | 1,754 | 24,750,000 | 5,763,000 |
| **Western Europe** | 3,000 | 650 | 13,000,000 | 2,500,000 |
| Belgium | 0.03 | 0.01 | 204 | 97 |
| France | 3,250 | 625 | 12,460,000 | 2,440,000 |
| Germany | 30 | 6 | 33,900 | 6,436 |
| Netherlands | 4 | 1 | 32,780 | 9,394 |
| Saint Pierre & Miquelon | 33 | 16 | 87,080 | 41,490 |
| **Oceania** | 84,000 | 23,000 | 257,000,000 | 65,000,000 |
| **Australia & New Zealand** | 19,000 | 4,800 | 85,000,000 | 19,000,000 |
| Australia | 5,430 | 1,040 | 53,490,000 | 10,210,000 |
| Lord Howe Isl. | 4 | 2 | 22,740 | 10,790 |
| Christmas Isl. | 3 | 1 | 16,110 | 7,650 |
| Cocos (Keeling) Isl. | 10 | 5 | 46,940 | 22,430 |
| New Zealand | 13,200 | 3,750 | 31,110,000 | 8,879,000 |
| **Melanesia** | 46,000 | 12,800 | 127,000,000 | 34,200,000 |
| Fiji | 12,900 | 2,460 | 51,481,000 | 9,817,000 |
| New Caledonia | 2,460 | 1,170 | 12,090,000 | 5,748,000 |
| Papua New Guinea | 18,000 | 5,140 | 35,730,000 | 10,110,000 |
| Solomon Is | 9,200 | 2,600 | 23,410,000 | 6,678,000 |
| Vanuatu | 3,090 | 1,470 | 3,933,000 | 1,879,000 |
| **Micronesia** | 12,000 | 3,700 | 23,000,000 | 7,100,000 |
| Guam | 8 | 4 | 40,920 | 19,400 |
| Kiribati | 7,580 | 2,150 | 8,121,000 | 2,303,000 |
| Marshall Isl. | 1,250 | 590 | 2,129,000 | 1,018,000 |
| Micronesia | 2,430 | 690 | 9,717,000 | 2,770,000 |
| Nauru | 161 | 77 | 322,600 | 154,600 |
| North Marianas | 20 | 10 | 61,890 | 29,630 |
| Palau | 758 | 216 | 2,886,000 | 824,000 |
| **Polynesia** | 7,000 | 1,600 | 22,000,000 | 4,800,000 |
| American Samoa | 12 | 6 | 50,880 | 24,010 |
| Cook Islands | 125 | 59 | 552,900 | 263,200 |
| French Polynesia | 2,880 | 550 | 13,190,000 | 2,518,000 |
| Niue | 32 | 15 | 102,500 | 48,700 |
| Norfolk Isl. | 1 | 1 | 8,054 | 3,847 |
| Pitcairn | 1 | 0.3 | 984 | 466 |
| Samoa | 2,760 | 520 | 5,415,000 | 1,026,000 |
| Tokelau | 82 | 39 | 128,300 | 61,330 |
| Tonga | 722 | 137 | 1,323,000 | 251,000 |
| Tuvalu | 288 | 138 | 301,200 | 143,900 |
| Wallis & Futuna Isl. | 179 | 85 | 960,300 | 461,300 |
| **Globally** | **2,925,000** | **835,000** | **5,587,000,000** | **1,529,000,000** |
