# Supplementary material for: Valuing invisible catches: Estimating the global contribution by women to small-scale marine capture fisheries production
Source: PLoS One. 2020 Mar 4;15(3):e0228912. doi: 10.1371/journal.pone.0228912 (PMC7055739; doi:10.1371/journal.pone.0228912)
Supplement: S1 Appendix — (DOC) [file pone.0228912.s006.doc]

# S1 Appendix: Country estimates, assumptions and uncertainty scores

**Africa**

**Eastern Africa**

1. **Madagascar**

In Madagascar, women play a dominant role in exploiting the rich mangrove ecosystems, selling products from traditional fishing, and working in processing factories (Monfort 2015). Women are key players in the valuable octopus fishery, representing the majority of those involved in the capture of octopus. However, traditionally this fishery was almost exclusively done by women, whereas today both men and women target octopus for subsistence and artisanal purposes but often using different methods and operate in different habitats (Westerman and Benbow 2013). Women glean for octopus in intertidal areas, whereas men dive in subtidal areas. Women also collect other invertebrates through intertidal gleaning (Barnes and Rawlinson 2009).

Surveys conducted in the Velondriake region of Southwest Madagascar found that 97% of fishers were men, 98% of gleaners were women, and 95% of those practicing both fishing and gleaning activities were men (Barnes-Mauthe *et al.* 2013). While this study collected data from the Velondriake region, the sample was extrapolated to produce a national estimate of the number of small-scale fishers and gleaners for Madagascar, which were both approximately 122,000 individuals (see ‘Supplementary Information’ in Barnes-Mauthe *et al*. 2013). This same study indicated that the same number of people were engaged in fishing as in gleaning, while an additional 24,000 individuals participated in both fishing and gleaning activities (i.e., 20% of the number of fishers). Gleaners, which were predominantly women, target octopus, sea cucumber, shellfish, and crab, with an estimated annual catch volume of 57,300 tonnes (Barnes-Mauthe *et al.* 2013). Whereas, fishers, of which 3% were women, had a catch volume of over 297,000 tonnes in 2010.

**Estimate:** Participation by women in small-scale marine capture fishing activities is 46% of small-scale fishers when including fishers, gleaners, and fisher-gleaners, together estimated to be approximately 268,800 individuals.

**Uncertainty:** The studies used for this estimate indicated high uncertainty associated with the data, in part because the surveys were done in only one region of the country and extrapolated to a country-wide estimate. However, similar estimates for small-scale fishing activities from this peer-reviewed publication were also found in a more recent census conducted by Blue Ventures for the same region (R. Singleton, pers. comm., November 24, 217). Therefore, with medium agreement and medium robustness, the estimate for participation in fishing activities receives a score of 2.

1. **Mozambique**

In the marine capture value chain of Mozambique, production activities mostly involve men, whereas women are more involved in post-harvest activities (Brugère and Maal 2014). Women bring fish from the landing sites to the markets, playing a key role in the distribution and sale of fish products as intermediaries (sometimes referred to as *maguevas* (Johnstone 2003; FAO 2008b). While the role of women as traders and vendors is more widely acknowledged, women also play a role in production through the inter-tidal collection of invertebrates for commercial and subsistence purposes (Johnstone 2003). Although women rarely go out fishing on boats, more and more women own boats, with 25% of the boatowners in the Bay of Maputo being women (Johnstone, 2003).

Fisheries landings from Mozambique’s Exclusive Economic Zone are predominantly from the small-scale sub-sector, which includes subsistence catches taken by women (Jacquet *et al*. 2016). In addition to the approximately 70,000 small-scale boat-based fishers in Mozambique (FAO, 2008), who are likely all male, there are approximately 52,777 shore-based collectors (Jacquet *et al.* 2010; Gervásio 2014), which includes women and children collecting invertebrates and small fish in the intertidal zone by hand, using spears, and with beach seines (Tietze *et al.* 2011).

**Estimate and Assumptions:** Participation by women in small-scale marine capture fishing activities is 28% out of small-scale 122,777 fishers including boat-based fishers and shore-based collectors. Assuming women are represented in the estimate of shore-based collectors described by Jacquet *et al.* (2010), this percentage of female collectors is assumed to similar to neighboring Tanzania (66%).

**Uncertainty:** This estimate receives a score of 1 for uncertainty. While several sources indicated that women participate in extraction activities, quantitative data were very limited.

1. **Tanzania**

Fishing in Tanzania is generally considered a male domain and, while very few women go out in boats, they participate in a dominant way in the pre- and post-harvest sectors and in fishing activities from shore (Matthews *et al.* 2012; Fröcklin *et al.* 2013, 2014; de la Torre-Castro *et al.* 2017). In the past, octopus fishing in Tanzania was an activity dominated by women and children. However, as the demand for octopus increased in the 1990s and the fishery became more profitable, men began participating in this fishery, displacing women (Guard and Mgaya 2002; Porter *et al.* 2008). As shore-fishers in mangroves, estuarine, and other nearshore areas, women collect sea cucumbers, crustaceans, and bivalves by foot using their hands or a wooden stick, while men use harpoons and spears (Marshall *et al.* 1999; Jiddawi and Öhman 2002; Silva 2006). Using a shore fishing catch rate of 0.97 t·year^-1^ (Jiddawi and Öhman 2002), approximately 4,000 tonnes of subsistence catch per year can be attributed to women.

**Estimate and Assumptions:** Participation by women in small-scale marine capture fishing activities is 8.7% out of a total of 49,500 small-scale fishers including shore-based and boat-based fishers. Based on sources that indicate that shore-based fishing activities are most often performed by women and that women dominate marine invertebrate harvesting from shore (Marshall *et al.* 1999; Guard and Mgaya 2002; Jiddawi and Öhman 2002; Silva 2006; de la Torre-Castro *et al.* 2017), participation by women in this fishery sub-sector was assumed to be 66% of collectors in Tanzania and Zanzibar (see Jacquet *et al.* 2010; Bultel *et al.* 2015). The number of shore-based fishers was calculated from a shore-based fisher to population ratio presented in Bultel *et al.* (2015). The result was a total of 6,500 shore-based fishers, which was added to the approximately 43,000 artisanal fishers, assumed to be boat-based fishers described in Jiddawi and Öhman (2002).

**Uncertainty:** This estimate received a score of 2, as quantitative data were limited, and the participation estimate was derived using a combination of sources.

**Middle Africa**

1. **Angola**

The marine fisheries sector in Angola consists mainly of industrial, artisanal, and subsistence sub-sectors, with a small recreational sub-sector (Belhabib *et al.* 2016a). Coastal communities in Angola rely on fishing and related activities as a key livelihood strategy, with both men and women participating in this sector (Raemaekers and Sunde 2015). Women are mostly involved in the post-harvest sub-sector, including buying fish from the boats when they come to shore, cleaning, and processing fish, as well as the sale of fresh, salted, and cooked fish. Some women are involved in subsistence fishing along the coast, but their numbers are not known (D. Belhabib, pers. comm. January 12, 2018).

**Estimate and Assumptions:** Participation by women in small-scale marine capture fishing activities is 0.5% out of approximately 18,000 small-scale fishers in Angola (Belhabib *et al*. 2015). This estimate assumed that participation by women in small-scale fishing activities was limited but not zero.

**Uncertainty:** The female participation rate receives an uncertainty score of 1 as there is a high degree of uncertainty with no quantitative estimate of female participation in fishing for this country.

1. **Cameroon**

There is a distinct gender division of labour in Cameroon, with men fishing and women processing and marketing the fish (Ngo Som 1995). Smoking and drying are the most common processing techniques in Cameroon, and these are performed almost entirely by women, specifically the wives of artisanal fishermen (FAO 2007a). Brummett *et al*. (2010) describe a traditional women’s fishery in freshwater bodies of southern Cameroon; however, accounts of female participation in marine fisheries were not found in the literature.

**Estimate and Assumptions:** Participation by women in small-scale marine capture fishing activities is estimated at 5% out of a total of 22,700 small-scale fishers (Belhabib *et al.* 2015). We assumed a participation rate in subsistence fishing activities similar to neighboring countries, so a rate of 5% was applied, based on the subregional average for West Africa.

**Uncertainty:** The uncertainty scores for fishing was 1, as there was very limited evidence for participation by women in fisheries for this country.

1. **Gabon**

Fishing in Gabon involves mostly men, while processing, marketing, and trading of fish is done mostly by women (Matthews *et al.* 2012). While the majority of fishing is done by men, women do participate in gleaning activities, collecting small fish and invertebrates from shore (Matthews *et al.* 2012) and catching shrimp in estuaries using small nets (FAO 2007b).

**Estimate and Assumptions:** Participation by women in small-scale marine capture fishing activities is 5% out of a total of 96,300 small-scale fishers (Belhabib *et al.* 2015). Female participation in fishing is assumed to be small but not zero as two sources indicate some level of extractive activity by women. As there was no available estimate for other countries in this subregion, female participation in fishing was assumed to be similar to neighboring countries, so the subregional average for West Africa of 5% was applied.

**Uncertainty:** This estimate received an uncertainty score of 1.

**Northern Africa**

1. **Algeria**

Marine fisheries in Algeria are dominated by industrial and artisanal sub-sectors, but also include some recreational and subsistence fisheries (Belhabib *et al.* 2016b). Fishing in Algeria is considered a male domain; therefore, women rarely participate in fishing activities. West African Fisheries expert, Dyhia Belhabib, who is also Algerian, indicates that “fishing is not regarded as a "noble" activity for a woman” and summarizes the following gender aspects of fisheries sector. There was one woman who operated a fishing vessel east of Algiers (Thalassa 2006) and another woman who fished commercially in the west, but neither is practicing today, with the latter now being a shipowner. The subsistence sector takes the form of men fishing with handlines from the beach.

**Estimate and Assumptions:** Participation by women in small-scale fishing activities is zero out of 10,000 small-scale fishers (Teh and Sumaila 2013). This estimate was based on personal communications from fisheries experts who indicated that participation by women in the fisheries activities is negligible.

**Uncertainty:** Estimates of female participation in fishing receives a score of 2 as there are several sources indicating women do not participate in fishing activities.

1. **Libya**

Participation by women in the fisheries sector is almost exclusively in processing (Reynolds *et al.* 1995). Of the approximately 11,500 fishers (FAO 2005b), there is no indication that any of these are women.

**Estimate and Assumptions:** Participation by women in small-scale marine capture fishing was assumed to be zero out of 11,500 fishers (FAO 2005b). Although no reference was found indicating that women are excluded from extractive fishing activities, Reynolds *et al.* (1995) indicate that women do not participate directly in fishing and are restricted to the processing sector; therefore, the assumed female participation rate in marine capture fishing is zero.

**Uncertainty:** This estimate receives a score of 1 as information on women in fisheries was almost non-existent for Libya with only one brief mention in Reynolds *et al.* (1995).

1. **Morocco**

In the Moroccan fisheries sector, women work almost exclusively in processing activities (FAO 1994). Women do not participate in artisanal fishing and other economic activities as dictated by socio-cultural structure of the region (ArtFiMed 2009). The marine capture fisheries sector employs approximately 176,000 of which roughly 50,000 are in fishing-related jobs; however, there is no indication of women in direct fishing activities.

**Estimate:** Participation by women in small-scale marine capture fishing activities was assumed to be zero out of 115,000 fishers (FAO 2005a).

**Uncertainty:** This estimate receives an uncertainty score of 1, given the limited quantitative information available on female participation in fishing activities.

1. **Tunisia**

In Tunisia, the participation by women in fishing activities focuses on the intertidal harvest of wild clams (ArtFiMed 2009; Gueye 2016; Ogden 2017). The clam production sector in Tunisia employs more than 4,000 women at 17 different harvest sites, primarily from two coastal areas – Gabès and Sfax, with an average annual production of 700 tons mostly directed for export (FAO 2017). The species targeted is *Ruditapes decussatus*, the Mediterranean wild clam. Women are also involved in the processing of the clams at facilities, which prepare the product for export to Europe (Gueye 2016), but estimates of the number of women participating in this aspect of the value chain were not found in the literature.

**Estimate:** Participation by women in small-scale marine capture fishing activities is 10% (this is based on 4,000 women out of a total of 41,270 small-scale fishers in Tunisia with the total number of fishers taken from an FAO estimate of the number of participants in the artisanal coastal fishery and the lagoon and foot-based fisheries) (FAO 2005d).

**Uncertainty:** This estimate receives a score of 3 as several references indicate that women are strong participants in the clam fishery, citing estimates, while the robustness of the estimate is considered medium as the source is reliable but from more than 10 years ago.

**Southern Africa**

1. **Namibia**

There is some evidence for participation by women in small-scale fishing activities in Namibia, using mosquito nets and collecting by hand from in flood plains, but these are likely only freshwater habitats (FAO 2007c). In terms of marine species, women collect shells along the Namibian coastline to make jewelry, known as *onyoka*. These are a traditional necklace made from mussel shells, commonly worn amongst the Oshiwambo speaking people. This activity has provided a means for many women to generate an income (Raemaekers and Sunde 2015). However, the collection of these shells is likely not considered under fisheries management as it involves only the empty shells. Nevertheless, this activity has an important socio-economic component that should be considered in overall economic assessments of marine resource extraction activities. However, since this is concerned with the removal of biomass from the ocean, here, female participation in fishing is zero.

**Estimate and Assumptions:** Participation by women in small-scale marine capture fishing activities is zero out of a total of 200 small-scale fishers. Assuming the description of women in fisheries given in the FAO country profile applies to freshwater environments, evidence from local experts suggests women are not involved in marine capture extractive activities.

**Uncertainty:** This estimate received a score of 1 as there was very limited information on female participation in fishing.

1. **South Africa**

Both men and women in South Africa are involved in various fisheries related activities, but data describing participation in these activities are limited (Harper *et al.* 2017). Participation by women in fishing activities is highlighted in a detailed study by Branch et al. (2002), which suggests that 20% of small-scale fishers in South Africa are women. Women mainly target oysters (*Striostrea margaritacea* and *Saccostrea cucullata*), mussels (*Perna perna*), and limpets (*Patella* spp and *Fissurella* spp) using knives, files, hoes and axes for subsistence and commercial purposes (Branch *et al.* 2002).

**Estimate:** Participation by women in small-scale marine capture fishing activities is 20% out of a total of 29,233 small-scale fishers (Branch *et al.* 2002).

**Uncertainty:** This estimate received a score of 3 as, although the data source was a detailed peer-reviewed study, it was done over a decade ago.

**Western Africa**

1. **Ghana**

Woman play important roles the Ghanian fisheries value chain, linking fishermen to consumers (Tetteh 2007). In Ghana, men go fishing and their wives and daughters process, transport, and market the fish. Strict rules and cultural beliefs prohibit women from setting foot in a canoe (Walker 2001; Failler *et al.* 2014). However, women at times fish by baskets and nets in lagoons (I. Issifu, pers. comm., May 1, 2019). Women dominate processing and marketing aspects of the fish value chain. Traditionally, men never assisted women in post-harvest activities; however, more recently some men are helping with processing, marketing, and distribution activities (Sasu 1999). Women are also key in financing fishing activities, raising capital by leveraging their social networks and status, and as owners of the means of production, such as fishing boats and processing equipment (Tetteh 2007; Gueye 2016). For example, in the Fanti town of Anomabo, 38% of canoes are owned by women (Walker 2002), while in the fishing village of Moree, an estimated 100 out of 400 canoes (25%) are owned by women (Overå 1992).

**Estimate and Assumptions:** Participation by women in small-scale marine capture fishing activities is estimated to be 2.5% out of approximately 12,000 small-scale fishers (Teh & Sumaila 2013). As there is evidence for women’s participation in lagoon fishing, but quantitative data was unavailable at the time of the analysis, we assumed a female participation rate that is half that of the subregional average for western Africa of 5%.

**Uncertainty:** This estimate receives an uncertainty score of 1 as it was based on an adjusted subregional average.

1. **Nigeria**

In Nigeria, women are present throughout the fish value chain with crucial roles in fish production, processing, distribution, and marketing (Williams 1996; FAO 2007d; Nwabeze *et al.* 2013; Cliffe and Akinrotimi 2015). Women play a significant economic role in the fishing communities of Nigeria, making important contributions to family income and to nutritional security (Williams 1996; Williams *et al.* 2005; Nwabeze *et al.* 2013).

While both men and women participate in fishing activities in Nigeria, men work on large boats, fishing in large and deep-water bodies, and women fish from smaller boats and canoes, and wade along the shores collecting shellfish and seaweed (Nwabeze *et al.* 2013). Throughout Nigeria women in fishing communities also use traps and nets to catch fish (Williams, 2006 *in* Okeowo *et al.* 2015). Women do not participate in deep sea fishing (Cliffe and Akinrotimi 2015), but they do participate in inshore fishing activities, including the brackish water canoe fishery that occurs in coastal creeks, lagoons, and mangroves in Nigeria. Species targeted in this fishery include catfish (*Arius* and *Chrysicthys* spp.), tilapia, mullet, and shellfish—such as shrimps (*Macrobrachium* spp.), crabs (*Callinectes* spp.), periwinkles, oysters and large quantities of the white shrimps, *Nematopalaemon hastatus*, commonly called “crayfish”, and penaeid shrimps (FAO 2007d).

Several case studies highlight the diverse roles of women in both freshwater and marine fisheries; however, at the country level, data on the number of women in the fisheries sector was not readily available. Cliffe and Akinrotimi (2015) describe the role of women in fishing activities of Rivers State, Nigeria from a qualitative study of fisherwomen in 10 communities. The study indicated that of the 200 fisherwomen surveyed, 50% were involved in collecting shellfish, while 40% participated in setting traps in rivers and 10% setting traps in creeks. Over half of the women surveyed participated in marketing, 30% in active fishing, 15% acted as middlemen, and 10% were involved in processing (Cliffe and Akinrotimi 2015). While there is some recognition that women participate in fishing activities, their participation in on-shore activities associated with the fisheries sector is better acknowledged with high female participation in this part of the fisheries value chain (Nwabeze *et al.* 2013).



**Estimate and Assumptions:** Participation by women in marine capture fishing activities is 12.8% (average female participation in a survey of artisanal fishers in two communities in Lagos State, Nigeria (Okeowo *et al.* 2015). Total number of small-scale fishers in Nigeria is estimated at 168,000 (Belhabib *et al.* 2015). In the absence of a country-wide estimate of participation by women in marine harvest activities, the survey by Okeowo *et al.* (2015) of female participation in artisanal fisheries for Lagos State was used as the best available estimate for participation in small-scale fishing.

**Uncertainty:** This estimate receives a score of 3 as several sources indicate that women participate in fishing activities, and one source provided a quantitative estimate from a case study, which was then scaled up to a country-level estimate.

1. **Senegal**

In Senegal, men dominate fish production activities while women control much of the post-harvest portion of the fish value chain (Soumare 2006). Most of the literature highlighting women in Senegalese fisheries has focused on post-harvest activities, where an estimated 90% of the approximately 40,000 seafood processors are women (Deme *et al.* 2012). However, women are also involved in production activities through the collection of invertebrates from shore for subsistence and/or artisanal purposes (Grandcolas 1997; Walter 2006). An estimated 1,350 women are involved in these shore-based fishing activities in Senegal, contributing an estimated 10,000 metric tonnes/year to the total national catch (Belhabib *et al.* 2014), valued as 30.5 million 2015 constant US dollars (Harper *et al.* 2017). Seven different species were identified as the target of women harvesting shellfish in mangrove estuaries: *Murex cornutus* (sea snail), *Cymbium senegalensis* (sea snail), *Pugilina morio* (whelk), *Senilia senilis* (blood cockle/clam), *Crassostrea gasar* (mangrove oyster), *Tagelus adansonii* (razor clam), *Callinectes sapidus* (blue crab); (Carney 2017).

**Estimate:** Participation by women in small-scale marine capture fishing activities is 2.3% out of a total of approximately 58,150 small-scale fishers (Belhabib *et al.* 2015).

**Uncertainty:** This estimate receives a score of 3 as several sources indicate participation by women in fishing activities with one providing a quantitative estimate from a peer-reviewed source.

**Americas**

**Caribbean**

1. **Cuba**

Women are an important part of the fisheries sector in Cuba with 6,331 women out of a total fisheries labour force of 37,635 (INFOPESCA 2018), i.e. 17%. Women participate in all activities with approximately 25% of primary fishing activities conducted by women (FAO 2015; Williams *et al.* 2017)

**Estimate:** Participation by women in small-scale fishing activities is 25% out of a total of 9,969 (FAO 2015)

**Uncertainty:** This estimate received an uncertainty scores of 2 as several sources indicate similar numbers.

1. **Dominican Republic**

In the Dominican Republic, fishing is predominantly small-scale and boat-based activities are conducted almost exclusively by men (Herrera *et al.* 2011). According to the Latin American Network of Women working in the fisheries sector (NETWIF), there are two women who fish in the Laguna de Oviedo and two women with motorized yolas in the province of Samaná (INFOPESCA 2018). Women are more involved in shore-based activities that support the fishery sector, including processing and marketing of fish (Herrera *et al.* 2011). While it is not common for women to go fishing on boats, women often own fishing boats and gear (Grant 2004).

**Estimate:** Participation by women in small-scale fishing activities is 0.04% out of 10,000 small-scale fishers (FAO 2008a; INFOPESCA 2018)

**Uncertainty:** This estimate received an uncertainty score of 2, as data were limited.

1. **Jamaica**

Women are involved in various aspects of the fisheries-related economy of Jamaica, which is an important source of food and livelihood for many people on the Island. In 2015, of the estimated 23,786 reported fishers, an estimated 6% were female (FAO 2016). Women occupy formal and informal positions in fisheries-related sectors and activities including investing, processing, marketing, and distribution, while also participating in fisheries associations and cooperatives (Grant 2004). The 1998 Jamaican Fisheries Census included a gender breakdown of vessel ownership, indicating that approximately 4% of fishing vessels were owned by women, with proportion of female-owned vessels being highest for mechanized fiberglass vessels <35ft (Grant 2004).

**Estimate:** Participation by women in small-scale fishing activities is 6% of 23,786 total registered fishers (FAO 2016).

**Uncertainty:** This estimate receives an uncertainty score of 2 as there was no explanation given for how the data were collected.

**Central America**

1. **El Salvador**

In El Salvador, both men and women participate in fisheries but often take on different roles and activities (Gammage 1996; OSPESCA 2012). Men fish the open seas, while women fish along the shoreline and in estuaries, targeting marine fish, crustaceans, and mollusks. Some women accompany other family members on open sea trips to catch shrimp, but women focus mostly on activities close to shore or onshore where they are disproportionately involved in processing the catch (Gammage 1996). A 2011 fisheries census for Central America indicated that out of a total of 27,600 artisanal fishers in El Salvador, 4200 were women, which translates to a female participation rate of 15.2% (OSPESCA 2012). Previous census data indicated that 6-9% of fishers were female, but these data are criticized for a methodology that may have overlooked many female participants (Gammage 1996). The more recent estimate was, therefore, assumed to be more accurate, although likely still underestimating the participation by women where census data often do not capture informal and unpaid labour contributions. For example, the many women involved in processing and marketing the catch at landing sites are not necessarily accounted for in the country’s employment statistics (FAO 2005c).

**Estimate:** Participation by women in small-scale fishing activities is 15.2% out of all total of 27,600 artisanal fishers (OSPESCA 2012).

**Uncertainty:** This estimate was assigned an uncertainty score of 2.

1. **Mexico**

In Mexico, fishing is culturally constructed as masculine work; however, women participate in various activities along the fish value chain, with the seafood industry relying heavily on temporary, part-time, and low-cost processing labour provided by women (Salazar and Castañeda 2002). A recent national census report suggested that approximately 10,500 women participate in fisheries in Mexico, mainly in processing and trade, representing 7% of total participation in fisheries (INEGI 2011). Additional sources highlight participation by women in the harvest of invertebrates (Mackenzie 2001; Valdez-Gardea 2001); however, quantitative details are rarely reported, except in Mackenzie (2001).

**Estimate and Assumptions:** Participation by women in small-scale fishing activities is 0.2% out of a total of 135,134 small-scale fishers (Harper *et al.* 2017). The only reference available regarding participation in direct marine capture activities was based on a regional study of cockle fishers (Mackenzie 2001). Several references point to minimal participation by women in direct capture activities; however, there is likely to be some additional participation by women in the collection of invertebrates from shore, as described in (Valdez-Gardea 2001).

**Uncertainty:** This estimate receives an uncertainty score of 1, due to extremely limited data sources and low agreement among sources.

1. **Panama**

While the role of women in fisheries in many Central American countries is not well documented, evidence does exist suggesting that women contribute in important ways to the fisheries economy of Panama. For example, in Pedro Gonzales, Las Perlas Archipelago (Panama) women participate in cleaning, filleting, drying, and cooking fish, as well as collecting shells and cleaning mollusks (Raab and Roche 2005). Beyond shellfish collection, women rarely take part in fishing activities according to Raab and Roche (2005). However, a 2011 fisheries census for Central America indicated that out of a total of 20,300 artisanal fishers in Panama, 700 were women (OSPESCA 2012), which translates into a female participation rate of 3.4%.

**Estimate:** Participation by women in small-scale fishing activities is 3.4% out of a total of 20,300 artisanal fishers (OSPESCA 2012).

**Uncertainty:** The estimate for fishing received an uncertainty score of 2. Although this was a country-specific estimate, it was the only quantitative account for female participation in fisheries, and an explanation of the survey methodology was not given for the data source.

**North America**

1. **Canada**

In Canada, fisheries sector participation is partially reflected in the National Census, which includes sex-disaggregated data on fisheries-related occupations (Statistics Canada 2016). The most recent census indicates that women represent approximately 13.5% of those working on fishing vessels as fishermen/women, as vessel masters and deckhands (Statistics Canada 2016).

Census data present only part of the picture as they account only for certain categories of work and may not include all fishing activities and efforts, such as subsistence fishing activities. Indigenous communities across all three ocean coasts engage in fisheries for food, social, and ceremonial purposes in addition to participation in commercial fishing. Participation in these fisheries may not be identified as an occupation or enumerated in the census, and while Indigenous men might be more likely to work in commercial fisheries, Indigenous women from various communities across Canada are known to participate in subsistence fishing activities (Sloan *et al.* 2002; Turner 2003; Kafarowski 2006; Williams 2006). Given the large number of Indigenous and coastal communities in Canada, the actual number of participants, both male and female, in fisheries, when considering both formal and informal activities, is likely much higher than the census data indicate. For example, on the Pacific coast in the Province of British Columbia, the census indicates that 350 of the 2,200 people that have identified their occupation as, ‘fishermen/women’, are female (Statistics Canada 2016). However, in the community of Bella Bella, which is one of many First Nations communities along the Pacific coast, approximately 400 of the 600 participants in the 2017 commercial herring spawn-on-kelp fishery were women (P. Waterfall, pers. comm., Heiltsuk Tribal Council, November 10, 2017). This suggests that the census data do not provide a complete picture of fisheries sector employment and participation by women.

**Estimate and Assumptions:** Participation by women in small-scale fishing activities is 13.5% (STATCAN, 2016) out of approximately 19,000 people involved in small-scale fisheries (Teh and Sumaila 2013). As Canada does not distinguish small- from large-scale fishing operations and employment, the overall estimate of participation by women in fisheries was applied to the total small-scale fisheries employment numbers from Teh and Sumaila (2013). The federal fisheries agency in Canada does not distinguish small- from large-scale sectors; however, I assumed that women are mostly engaged in small-scale fishing activities.

**Uncertainty:** A score of 3 was given for uncertainty. While the data are derived from census data, other sources suggest that these are not comprehensive in their inclusion of the types of work in fisheries that often involve women.

1. **Greenland**

In Greenland, fishing is the primary industry supplying much of the employment to the island. Fishing is done mostly by men, with only 10 of the 2,716 commercial licenses held by women (Sloan *et al.* 2002). However, as in many other countries and contexts, women provide considerable support to fishing businesses and operations, with important social and economic roles in fishing communities (Ford and Goldhar 2012).

**Estimate and Assumptions:** Participation by women in small-scale fishing activities is 0.4% out of a total of 2,716 commercial fisheries licenses holders in 2003 (Sloan *et al.* 2002). License holders are roughly equivalent to the number of participants.

**Uncertainty:** Given the limited availability of data that is both recent and comprehensive in the categories it includes, the uncertainty score for this estimate is 2.

1. **United States**

While numerous sources indicate that fishing in the United States is mostly a male domain, especially in terms of activities on the water, women are involved in the broader fisheries-related economy in important ways, particularly in terms of shore-based support to fishing operations and businesses (Reedy-Maschner 2009; Calhoun *et al.* 2016). In many parts of the United States small fishing businesses have long relied on women providing logistical support to fishing operations, e.g., cooking for the crew, distributing paychecks, picking up parts, and taking care of the financial side of the business (Calhoun *et al.* 2016). While the role of the fishermen’s wife has long been recognized, in terms of contributions to the economy, these roles are rarely captured in census and statistical accounts of labour contributions, as these are not necessarily formal or paid positions but contribute substantially to the fisheries sector. Some activities, such as bookkeeping in small fishing businesses, is often done by women and is simply considered a family responsibility, while in other cases, women may run bookkeeping businesses and may charge a fee for this service (Howell 2002).

Overall, women are thought to represent a very small proportion of on-the-water fisheries sector participation, with some estimates indicating that women represent less than 1% of commercial fishermen; however, there is likely considerable regional variation; for example more women go out fishing on the US west coast fisheries than on the east coast (Howell 2002), and in Alaska, it is not uncommon for women to work as commercial fishermen and observers on boats (Laukitis 2017).

Census data in the United States does not provide the level of detail necessary to determine the number of women involved in small-scale marine capture fishing activities and in fisheries-related activities, such a fish processing. The best available estimate of female participation rates in the fisheries sector is the percentage of women in the occupation category, ‘fishing, farming and forestry’. It is possible to exclude agricultural work from this estimate to get an overall female participation rate for fishing, forestry and hunting, which in 2016 was 9.4% (U.S. Census Bureau 2016a). This is the closest available approximation for participation by women in fisheries at a National level. The only other estimate of relevance is from the National survey of fishing, hunting and other wildlife-related recreation, which indicated that, in 2016, approximately 27% of recreational anglers in the US were female, and there were an estimated 8.3 million recreational saltwater anglers across the United States (US Census Bureau 2016b).

**Estimate and Assumptions:** Participation by women in small-scale fishing activities is 9.4% (US Census Bureau 2016a) out of a total of 29,000 fishers and related fishing workers (US Bureau of Labor Statistics 2017). In the absence of data disaggregated by sector, the female participation rate for the aggregated category ‘fishing, hunting and forestry’ from census data as representative of female participation in fishing was used.

**Uncertainty:** This estimate receives a score of 2 as it was based on a broad census category that included other sectors that may not have a similar gender composition as for fisheries.

**South America**

1. **Brazil**

Women participate in the fisheries-related economy of Brazil in many ways, although their roles and activities are not necessarily recognized (Rocha and Pinkerton 2015; Ferrari 2016; Alonso-Población and Siar 2018). There are approximately one million registered artisanal fishers in Brazil, of which 45% are women (Ferrari 2016), with the majority located in the North and Northeast of the country (FAO 2010a). In the state of Bahia, over 20,000 women participate in shellfish collection as *marisqueiras*, while in the state of Maranhão, women capture shrimp from shore using small nets, a practice that also occurs in other Brazilian states (Diegues 2008). Women also participate in shrimp, crab, and mollusk fisheries in the south of Bahia (Di Ciommo 2007). On the Northeast coast of Brazil (Ponta do Turbarão), Venus clam (*Anomalocardia brasiliana*) harvesting is an activity dominated by women (i.e., two thirds of active clam harvesters were women, accounting for 80% of the registered clam harvest activity (Rocha 2013; Rocha and Pinkerton 2015). Men also participate in the harvest of Venus clams but only as a secondary fishing activity, when other target species were unavailable or in times of economic need.

**Estimate and Assumptions:** Participation by women in small-scale fishing activities is 45% out of a total of one million artisanal fishers (Ferrari 2016). This number reflects an expanded definition of fishing, which was then narrowed following legislation changes that occurred in 2015 (Ferrari 2016). However, I assumed that this was the most comprehensive estimate of fishing activities, accounting for the many women that participate in the direct extraction of marine resources as shellfish harvesters.

**Uncertainty:** The estimate of female participation in fishing activities receives an uncertainty score of 3 as this was based a recent estimate of national coverage that was inclusive of a range of fishing activities.

1. **Chile**

Women are an important part of the fisheries sector in Chile; however, the contributions by women in fisheries are only recently being recognized and accounted for in fisheries statistics. In 2004, efforts were made in Chiloe Province to make visible the formal and informal contributions by women to the fisheries sector (Araneda *et al.* 2005), and while this study suggests that in the past women have not been well accounted for in fisheries statistics, more recent efforts at enumeration likely better reflect the contributions by women in the fisheries sector. The most recent fisheries census data from the National fisheries and aquaculture agency, SERNAPESCA, indicated that in 2017 women make up approximately 23% of artisanal fishers, a category which includes divers, shore collectors, algae harvesters, and artisanal shipowners in addition to more traditional definitions of fishing (Berazaluce Maturana *et al.* 2017).

**Estimate:** Participation by women in small-scale fishing activities is 23% out of a total of 86,056 marine coastal fishers (Berazaluce Maturana *et al.* 2017).

**Uncertainty:** The estimate of participation in fishing activities receives a score of 4, as the estimate was from a comprehensive survey with national coverage, was recent, and was from a robust source.

1. **Peru**

While the fisheries sector in Peru is often considered a male domain, women play an important role in the fisheries-related economy, but their work often goes unrecognized (Delgado-Gustavson 2011). The success of fishing businesses and operations in Peru relies heavily on the unpaid work that women perform to maintain the household and community networks (Delgado-Gustavson 2011). Women also participate directly in marine fisheries extraction through the collection of bait (Garcia 2000), invertebrates (Silva 2000), kelp, or small fish from shore. Rarely do women go out on boats and never for more than a day; the industrial vessels are almost entirely crewed by men. Women often work at landing sites doing the initial/primary processing of cleaning, gutting, and cutting fish (S. de La Puente, pers. comm., University of British Columbia).

A recent census, for the first time, provided quantitative estimates of female participation in the fisheries sector, with approximately 1,350 women engaged in artisanal fishing activities (representing just over 3% of artisanal fishers) and another 2,050 women as artisanal vessel owners (representing 16.5%: Instituto Nacional de Estadística e Informática 2012). The census also included sex-disaggregated data on species targeted and gear used by men and women. Women's participation in fisheries is concentrated in the south of Peru, mainly because of the migration of people from the Andes during political conflicts that took place in the late 1980s and 1990s. Artisanal vessels are often owned/registered under women's names, through joint-ownership (S. de La Puente, pers. comm., University of British Columbia).

**Estimate and Assumptions:** Participation by women in small-scale fishing activities is 4.6% out of a total of 44,161 artisanal fishers/shipowners (Instituto Nacional de Estadística e Informática 2012). Here, ship owners are included in estimates for participants in the primary sector; however, since shipowners might also be fishers, the potential overlap between the 1350 female fishers and the 2046 female ship owners was accounted for in the calculation. The female participation rate in small-scale fishing activities was estimated by dividing 2046 by the total number of small-scale fishers, assuming full overlap in the case of male fishers to be conservative. Secondly, it was conservatively assumed that the female participation rate for the artisanal sub-sector was similar for the subsistence subsector.

**Uncertainty:** This estimate received an uncertainty score of 4, as this was from data collected recently as part of an artisanal fisheries census covering the entire country.

**Asia**

**Eastern Asia**

1. **China**

Gender roles in fisheries changed substantially in China when the state removed the prohibition on women going to sea in 1958 and with the economic reforms to the country which started in the late 1970s (Xu *et al.* 2012). As women started to occupy new economic spaces, many entered the fisheries workforce. Today, women are involved in all stages of small-scale fisheries production, including in fish processing, preservation, and marketing, but constitute a higher proportion of the labour force in fish processing and distribution (Wang and Zhou 2008). Women represent approximately 22% of the reported fishers in 2013 (e.g., 869,699 women out of a total of 3,906,874 traditional fishers in China; Fisheries and Fisheries Administration of the Ministry of Agriculture 2014). Estimates from a World Bank study that were similarly derived indicate a female participation rate in the fisheries workforce of 19% for China (World Bank 2010).

**Estimate and Assumptions:** Participation by women in small-scale marine capture fishing activities is 22% of 1,454,571 fishers (Fisheries and Fisheries Administration of the Ministry of Agriculture 2014). It was not clear whether the number of people included in the Fisheries Yearbook for China included all those included in the fisheries sector or just those involved in fishing. Also, this estimate likely includes those involved both in large and small-scale sectors and in aquaculture. From the Fisheries Yearbook, approximately 60% of those involved in fisheries are in wild capture fisheries as opposed to aquaculture. A female participation rate of 22% was applied only to the portion of the fisheries that is considered small-scale.

**Uncertainty:** Fishing estimate receives an uncertainty score of 1 (high uncertainty), as although the estimate is based on national statistics, it is unclear how the data were collected, what exactly it includes/excludes, and whether this number refers only to fishing or also includes related activities.

1. **Japan**

The Japanese government provides detailed statistical records, available online in both Japanese and English, of the number of people participating in fisheries by gender and age (Ministry of Agriculture Forestry and Fisheries 2016). The most recent data indicate that in 2014 there were 22,580 women out of a total of 173,030 individuals engaged in coastal marine fisheries, which translates into a female participation rate of 13%. As there is no formal, legal definition of small-scale fisheries in Japan, these numbers have not been disaggregated into small- and large-scale sub-sectors. However, approximately 94% of the fisheries fleet in Japan are fishing boats smaller than 10 gross tons and are considered as small-scale vessels and fishers (Delaney and Yagi 2017). It is unclear as to whether the government statistics include all fisheries extraction activities, including Ama divers.

The ancient Japanese tradition of the Ama goes back at least 2,000 years, involving both men and women free diving for seaweed, shellfish, sea urchins, lobster, sea cucumber, oyster, octopus and abalone (Lim *et al.* 2012). The number of people who participate in this activity was estimated in 1989 at approximately 20,000, with a female participation rate of 41.2% (Lim *et al.* 2012).

**Estimate and Assumptions:** Participation by women in small-scale marine capture fishing activities is approximately 13% of 163,648 people engaged in small-scale marine capture fisheries. To disaggregate small- from large-scale fisheries, the fleet estimate of 94% was applied to the total number of people engaged in fishing, to represent participation in small-scale fisheries. To remain conservative, it was assumed that the fisheries census included Ama divers in the number of people engaged in fishing.

**Uncertainty:** The estimate for female participation in small-scale fisheries receives an uncertainty score of 3 as several sources indicate similar estimates of participation by women and the data were from a robust source covering the entire country (i.e. a recent labour force survey); however, it is unclear whether these numbers include all marine fisheries extraction activities and whether the collection of data was done in a way to avoid gender bias.

**Southeastern Asia**

1. **Indonesia**

Fisheries in Indonesia are characterized by a gendered division of labour, where men focus on production and women focus on post-harvest activities (Siason *et al.* 2002). However, a closer look reveals that women are engaged throughout the fish value chain and that “the complex economic networks for catching, selling, distributing and processing of the fish products often involve the women” (Anna 2012). The contribution by women to the fisheries-related economy is substantial and complex but not well recognized, (i.e., the work by women in fisheries is not counted in national government census collections under fisheries related employment), with the exception of a few studies that have focused on highlighting these overlooked contributions by women (Glaeser and Glaser 2011; Anna 2012; Fitriana and Stacey 2012; Matthews *et al.* 2012). For example, women on the Pantar Islands participate, alongside men, in small-scale capture activities, such as gleaning for molluscs along the intertidal zone and trapping small-fish in nearshore waters either on foot or using small motorized boats (Fitriana and Stacey 2012). Whereas in Aceh, local taboos prevent women from participating in fishing activities (Matthews *et al.* 2012). In East Kalimantan, Bajau women collect and trade giant clams from nearshore marine habitats on the Berau coast for subsistence and commercial purposes (Máñez and Pauwelussen 2016).

The only quantitative data on participation by women in fisheries located was from a report on the informal employment sector in Indonesia, which gave estimates of men and women employed in fishing for the Provinces of Yogyakarta and Banten, which had female participation rates of 35% and 2%, respectively (Asian Development Bank 2011).

**Estimate and Assumptions:** Participation by women in small-scale marine capture fishing activities is 10% out of a total of 2,169,279 engaged in marine capture fisheries (Asian Development Bank 2011; Fitriana and Stacey 2012). Assuming the estimates from the Asian Development Bank refer to fishing and not to post-harvest activities, such as processing and vending, which would fall under broader categories of employment, an average participation rate of 10% based on the survey of the two provinces is used as the estimate for female participation in marine capture activities

**Uncertainty:** For this estimate the robustness of the data and agreement were very low, meaning there is a high degree of uncertainty and, therefore, received a score of 1. The data used was from a survey of two provinces, with no clear definition of what was included in their definition of fishing.

1. **Malaysia**

Although traditions and superstitions in Malaysia limit the involvement of women in fisheries, women perform essential but underestimated roles in fisheries-related activities, such as unloading, sorting, gutting, net mending, processing, and distribution and marketing (Yahaya 2001). These roles vary by region; for example, in Peninsular Malaysia, women in the east coast states especially Kelantan, are more actively involved in the marketing of the catch than women in the west coast (Siason *et al.* 2002). In Sabah, East Malaysia, women glean during low tide and swim when the water is shallow to look for urchins and shellfish, which they either keep for home consumption or sell in small quantities at the market. Women also accompany their husbands out to sea for one to several nights to cook and help with boat tasks, and some of them also help with mending gear at home (L. Teh, pers. comm., March 13, 2018)

Women who participate in small-scale fisheries activities are often the wives or daughters of fishers and, in many cases, work to supplement family income or provide unpaid labour for home-based family businesses (Siason *et al.* 2002). There are instances where women do fish, with evidence from the east coast states of Kelantan and Terengganu and, to a lesser extent, Kedah on the west coast of Peninsular Malaysia. These "fisherwomen" fish mainly from the shore or in shallow protected waters using simple hand-operated gear, such as hooks and lines, scoop nets, or traps. The catch is used primarily for home consumption while surplus may be sold, traded, or gifted to local fish dealers, village retailers, friends, and relatives (Yahaya 2001).

In Malaysia, no census or documentation on the actual numbers of women involved in the various fishing activities has been carried out (Yahaya 2001). Participation by women in capture fishing is thought to be limited in Malaysia, with the exception of those using traditional small gear, such as the *bintoh* for catching crabs, fish traps, or *bubu* for catching fish, and some may accompany their husbands out to sea (Siason *et al.* 2002). Women fish from shore mainly for subsistence purposes.

**Estimate:** Participation by women in small-scale marine capture fishing activities is 17.5% out of a total of 51,480 coastal fishers (FAO 2009a).

**Uncertainty:** The estimate for female participation in fishing activities receives an uncertainty score of 1 as there was no quantitative data available that was specific to Malaysia, so a subregional average was used.

1. **Thailand**

Fishing in Thailand has typically been considered a male occupation; however, women participate in fishing activities but may not refer to themselves as fishers (Poonnachit-Korsieporn 2000). A fisheries census conducted in 2000 indicated that out of a total 80,538 people engaged in the marine capture fisheries sub-sector, 12,242 were women (National Statistical Office 2001), which translates into a female participation rate of 15.2%. This number likely underestimates female participation in fishing activities, which has been found to be higher in some regions. For example, a study of the Andaman Coast of Thailand found that one third of fisherfolk in the bay of Phang-nga were women. However, this study does not provide detailed descriptions of the roles and activities of female participants (Seilert and Sangchan 2001).

**Estimate and Assumptions:** Participation by women in small-scale coastal marine fishing activities is 15.2% out of a total of 69,665 people in small-scale coastal marine fishing activities based on census data. FAO (2009b) suggests that there are 800,000 people involved in the primary sector in fisheries. To disaggregate small and large-scale sectors, the estimate that 86.5% of fisherfolk are small-scale from Poonnachit-Korsieporn (2000) was used.

**Uncertainty:** This estimate receives a score of 2 as it was based on limited data which lacked detail on how numbers were estimated (not clear how comprehensive/gender inclusive). Additionally, data were from over a decade ago.

1. **Vietnam**

Fishing households in Vietnam are characterized by a gendered division of labour, with men going out to sea to fish while women engage in selling and processing fish (World Bank 2005). Approximately 1 million people engage directly in fishing activities (Than Thi Hien 2008). While there is evidence that women participate in marine capture fisheries (e.g., fishing from boats in lagoons and collecting invertebrates by foot from shore (World Bank 2005; Lentisco and Phuong Thao 2013), these activities are not well documented or accounted for. Than Thi Hien (2008) estimates that roughly 40,000 women are involved in marine capture fisheries, which translates into a female participation rate of 4% (Harper *et al.* 2017).

**Estimate and Assumptions:** Participation by women in coastal marine fishing activities is 4% out of approximately 1 million (Than Thi Hien 2008). The female participation rate cited in Than Thi Hien (2008) was assumed to represent participation in the small-scale sector, as several sources indicated that women do not participate in large-scale fishing activities.

**Uncertainty:** This estimate receives an uncertainty score of two as, although the numbers used to derive the estimate were from a peer-reviewed publication, the original source of the data was from an unpublished source.

**Southern Asia**

1. **Bangladesh**

In Bangladesh, fishing has traditionally been considered a male occupation; however, today, women contribute to the fisheries economy in many ways, including the post-larvae shrimp collection and in processing and marketing seafood, among other fisheries-related activities (Sultana *et al.* 2002; Rabbanee and Yasmin 2011). The 2013 labour force survey for Bangladesh indicated that 1,970 of a total of 136,372 persons engaged in coastal and marine fisheries were women (Bangladesh Bureau of Statistics. 2015). However, if we consider participation by women in marine extractive activities more broadly and include the majority of the 450,000 seasonal shrimp fry collectors which are women (FAO 2014), female participation in fishing would be much higher.

**Estimate and Assumptions:** Participation by women in small-scale marine capture fishing activities is 5% out of a total 760,100 peoples (FAO 2014). The various sources consulted indicated vastly different estimates for the number and/or percentage of women working in fisheries. This is likely due to differences in survey methodology as well as discrepancies in definition of the types of fisheries and work included. A female participation rate of 5% was applied to a total participation in marine capture fishing of 310,100 persons plus 450,000 fry collectors. This is a conservative estimate, considering the majority of shrimp fry collectors are thought to be women; however, as there was no quantitative estimate given for female participation in fry fishing, a lower participation rate was assumed.

**Uncertainty:** This estimate had high uncertainty as the data were not clearly explained, and some conflicting estimates existed; therefore, Bangladesh received a score of 2.

1. **India**

Women make significant contributions to the fisheries sector and related economy in India, occupying a range of roles (Ashaletha *et al.* 1995; Immanuel *et al.* 2003; Durai and Dhanalakshmi 2015). While roles may vary between fishing communities and regions depending on local culture, religious beliefs and other factors, in many parts of India women have traditionally been involved in the processing and marketing of the catch, in net making and repair, and in some cases in the operation of shore seines, the collection of shellfish and seaweed in tidal areas (Tietze *et al.* 2007). Women are also instrumental in the functioning of the fishing household, providing much of the unpaid labour necessary to run the household and family fishing operations, including tasks related to financial management, family welfare, provisioning for fishing trips, etc. (Ashaletha *et al.* 1995).

According to the 2010 Census from the Marine Fisheries Service in India, just over half of the fisherfolk engaged in fish seed collection in the mainland of India are female, and over 80% of fisherfolk engaged in marketing and processing of fish are women (Central Marine Fisheries Research Institute 2010). A separate census conducted for the Islands of Andaman & Nicobar and Lakshwadeep indicates that a quarter of seed collectors are women and over a third of fisherfolk engaged in allied fishing activities are women (Fishery Survey of India 2012). Estimates of those actively engaged in fishing are not disaggregated by sex and, while several sources indicate that women are not actively involved in fishing, some women have been observed actively fishing alongside male family members in the Middle Andaman’s (S. Advani, pers. comm., February 10, 2018). Women participate in fish seed collection and the shore-based collection of invertebrates (Immanuel *et al.* 2003; Immanuel and Rao 2009; Durai and Dhanalakshmi 2015), but these activities are not necessarily considered “fishing”; however, I considered these to be extractive activities and included them in my estimate for small-scale fisheries. Some regional estimates of participation by women in shore-based gleaning activities include an estimated 1,200 women of a total of 5,500 people involved in mussel and oyster collection in the Vembanad estuary in Kerala (Koshy and Sharma 2007). It is not clear to what extent these fishing activities are included in the National Marine Fisheries census, but a recent study suggests improved census data collection is needed to highlight the many women in the fisheries sector than are overlooked in the current system (Gopal *et al.* 2017).

**Estimate and Assumptions:** Participation by women in small-scale marine capture fishing activities is 4.6% out of a total 1,021,851 people engaged in fishing, fish seed collection, and shellfish gleaning are women (Central Marine Fisheries Research Institute 2010; Fishery Survey of India 2012). The Marine Fisheries Census (2010) includes a note associated in the table of allied fishing activities, which describes the category ‘other’ as, “Includes persons engaged in auctioning, ice breaking, collection of bivalves, collection of other shells, collection of seaweed, collection of ornamental fish etc.” This category was considered here as part of the marine capture fishing and was added to estimates of ‘other’, fish seed collectors, and active fishers for a more comprehensive estimate of participation in marine capture fishing activities.

**Uncertainty:** The estimates for female participation in fishing receives an uncertainty score of 3 as the data are from a relatively recent national census; however, it is very likely that the National Census (based on a frame survey) overlooks many of the informal and unpaid activities often dominated by women.

1. **Iran**

In Iran, as in other conservative Islamic countries, the culture and the state restrict participation by women in work outside the home (Siason *et al.* 2002). Employment for women in Iran is very limited, and fisheries is not one of the few occupations that women participate in (Alaedini and Razavi 2005).

**Estimate and Assumptions:** Participation by women in small-scale marine capture fishing activities is zero out of a total of 42,000 people working in small-scale fisheries activities (Teh and Sumaila 2013). There was very limited information on participation by women in the fisheries sector of Iran. It was assumed that women do not participate in either fishing or post-harvest activities, as this type of work is not recognized as acceptable for women.

**Uncertainty:** For participation in fishing, the estimate receives an uncertainty score of three as multiple, reliable sources suggest women do not participate in fisheries.

**Western Asia**

1. **Oman**

Fisheries in Oman are almost entirely small-scale and involve both men and women; however, women are not well recognized for their contributions. A recent study brought attention to women in this sector, highlighting their role in the capture, processing, and marketing of marine species (Al Rashdi and McLean 2014). This study showed that women are engaged in activities, such as gathering, gleaning, and spear fishing, for gastropods, bivalves, sea cucumbers, cephalopods, and crustaceans along the coast. While national fisheries statistics do not include estimates of fisherwomen, the study of the Al-Wusta Governorate found that 9% of fisherfolk in the region were women (Al Rashdi and McLean 2014). The study acknowledged that the communities surveyed may not be representative of participation by women in other regions of the country where women may not be as involved in fishing activities.

**Estimate and Assumptions:** Participation by women in coastal marine fishing activities is 3% out of a total of 45,000 small-scale fishers in Oman (FAO 2013). This assumes that the national female participation rate is approximately one third of the rate found in the study of Al-Wusta.

**Uncertainty:** This estimate received a score of 3 as, although the data were rigorously collected, from a peer-reviewed source, they only covered a portion of the country so were scaled up, using some assumptions to produce a country-level estimate.

1. **Turkey**

Women make important contributions to fisheries in Turkey, but their recognition as sectoral participants is relatively new with Turkish fisheries statistics (TÜİK) adding fisherwomen as a category only in 2012. According to TÜİK (2015), the female participation rate in fishing activities is approximately 1% for the entire country. However, focused studies have shown this proportion to be higher in some regions, i.e., 4.2% in the Southern Aegean (Göncüoǧlu and Ünal 2011) and as high as 20% on the Datça-Bozburun peninsula (Göncüoğlu *et al.* 2015). Moreover, Göncüoǧlu and Ünal (2011) indicated that only 38% of fisherwomen in the Aegean region are registered, which suggests that the actual number of women participating in fishing activities may be much higher than national statistics indicate. Women participate mainly in small-scale fishing activities, often as crew on boats with their husbands (Göncüoǧlu and Ünal 2011) but are also extensively involved in post-harvest activities such as processing.

**Estimate and Assumptions:** Female participation rate in marine capture fishing activities is 2% out of a total 37,747 people employed in coastal marine fisheries. The female participation rate was derived from an average between the two rates cited in the literature and under the assumption this was for the small-scale subsector.

**Uncertainty:** This estimate has a high degree of uncertainty and therefore received a score of 2. Although the estimate was taken from census data and was supported by peer-reviewed literature, the sources themselves identify gaps in accounting.

1. **Yemen**

In Yemen, women are not actively involved in fishing or marketing of fish but participate formally in the fisheries economy by way of seafood processing (Bonfiglioli and Hariri 2004).

**Estimate:** Participation by women in small-scale marine capture fishing activities is zero out of a total of 60,000 people actively engaged in small-scale fishing (Bonfiglioli & Hariri 2004).

**Uncertainty:** This estimate received an uncertainty score of 2, given the limited information.

**Europe**

**Eastern Europe**

1. **Poland**

In Poland approximately 60% of those employed in fishing are in the small-scale sector (STECF 2017). In terms of female participation, an estimated 2% of those employed directly in fishing are women (Salz *et al.* 2006).

**Estimate and Assumptions:** Participation by women in small-scale fishing activities is 2% out of a total of 1,397 people in small-scale fishing. The female participation rate of 2% from 2003 was applied to the estimated number of small-scale fishers in 2015, assuming female participation rates did not change considerably over that time period and assuming the women involved directly in fishing activities participated in the small-scale sub-sector.

**Uncertainty:** This estimate receives a score of 2 as the data were from more than a decade ago and likely only reflect permanent positions.

1. **Russia**

While it is mostly men that participate formally in fishing activities in Russia, women are involved in the informal and unpaid sectors (Zyalya Partal 2018).

**Estimate and Assumptions:** Participation by women in small-scale fishing activities is 2% out of a total of 20,000 small-scale fishers (Teh and Sumaila 2013). Given the very limited information on women in fisheries, the estimate of female participation rates in fishing was based on that of neighboring countries, assuming culturally similarities in terms of participation in the fisheries sector.

**Uncertainty:** This estimate received a score of 2, as it is based on a subregional average.

1. **Ukraine**

Fishing in the Ukraine is a male-dominated economic activity, while processing is a female-dominated activity (Libanova *et al.* 2012).

**Estimate and Assumptions:** Participation by women in small-scale fishing activities is 2% out of a total of 25,000 in fishing activities (Teh and Sumaila 2013). As there were no available data on female participation in fishing activities, a benefit transfer approach was used, applying the estimate from Poland.

**Uncertainty:** This estimate receives a score of 2 as the data was from a neighboring country.

**Northern Europe**

1. **Denmark**

In Denmark, women represent approximately 3% of registered fishers, whereas their participation in the processing sector is much, higher with women representing approximately half of seafood processors (Salz *et al.* 2006; Döring *et al.* 2012; OECD 2015).

**Estimate:** Participation by women in small-scale fishing activities is 3.6% out of a total of 1891 coastal fishers (OECD 2015).

**Uncertainty:** The uncertainty estimate is 3, as multiple sources indicated similar numbers, but there was minimal explanation of how the data were collected. It is unclear how comprehensive these estimates are.

1. **Norway**

Fishing in Norway has long been considered a male domain; however, women are involved in many roles and activities but are often not acknowledged for their broader contributions to the fisheries economy (Gerrard 2018). Recent data from 2016 indicate that approximately 3.1% of registered fishers in Norway are women, but this estimate does not include the many additional informal and unpaid inputs women make to fisheries. For example, women often act as “the shore crew”, providing support to fishing businesses and operations, and as household and community managers (Gerrard 2005). In the seafood processing sector, women represent a much higher percentage of the formal workforce. In 2011, there were approximately 813 women out of 2074 workers in the processing sector of Finnmark, which translates into a female participation rate of 39% (Neis *et al.* 2013). Overall, fishing and related jobs have decreased since the quota system was introduced in 1990, which has affected both men and women in the industry but with some gendered impacts that have resulted in further marginalization of women in the fishing industry (Munk-Madsen 1998; Gerrard 2006, 2018). While the rest of Norway is considered a hallmark for gender equality, the fishing industry lags in terms of both recognition and support of women in the industry.

**Estimate:** Participation by women in small-scale fishing activities is 3.1% out of 11,600 coastal fishers (OECD 2015; Gerrard 2018).

**Uncertainty:** This estimate received a score of 2 as this only includes formal employment/registered fishers, where there is additional, unaccounted for, participation in informal and unpaid activities.

1. **United Kingdom**

Women in the United Kingdom (UK) are involved in the fisheries-related economy in a variety of roles and activities, which are not necessarily visible or reflected in fisheries employment statistics (Zhao *et al.* 2013, 2014). A report on Women in European fisheries indicated that less than 1% of those participating directly in fishing were women (MacAlister Elliott and Partners LTD 2002). Similarly, Zhao *et al.* (2013), in their interviews of fisheries sector participants, found that only a handful of women went out fishing on boats or harvested cockles from shore, whereas many more women were involved in other fisheries-related activities.

**Estimate and Assumptions:** Participation by women in small-scale fishing activities is 1% out of a total of 11,800 (Zhao *et al.* 2013; Teh and Sumaila 2013). For this estimate, a female participation rate of 1% was used as there was evidence for women participating in this sector but qualitative descriptions indicate their involvement is limited.

**Uncertainty:** The estimate for female participation in fishing gets a score of 2.

**Southern Europe**

1. **Greece**

In Greece, the few women who work on fishing boats are there supporting their husbands as part of a family business (Quist *et al.* 2010). To become formally registered as fishers, men and women are required to have a diploma. In fisheries, women are much more prevalent in the processing sector with fish processing factories employing mainly women. In terms of unpaid work, women contribute substantially to the family-based fishing enterprise as spouses, mothers and sisters, looking after many important aspects of the fishing business including the financial management, communication with fishery administration, banks and book keepers, buying and delivering supplies to the boat and crew. By providing unpaid labour, these women aim to reduce business costs and to increase revenues as a way of contributing to the family, especially during times of financial crisis. Women also work in ancillary jobs associated with small-scale fisheries sector, such as making and repairing fishing gear (Quist *et al.* 2010).

In terms of recognition, women’s work is partially captured in fisheries employment statistics where their roles are formalized. The most recent sex-disaggregated data on participation in fisheries was a 2006 EU fisheries sector employment report, which suggested that women represented 6% of fishing employment and 50% of fish processing sector employment in Greece (Salz *et al.* 2006). In 2016 there were 24,759 people employed in fishing activities, 79% of which were associated with the small-scale sub-sector (STECF 2017). This estimate assumes that women working in productive or extractive activities are mainly in the small-scale subsector.

**Estimate and Assumptions:** Participation by women in small-scale fishing activities is 6% out of a total of a total of 19,560 people employed in small-scale fishing (Salz *et al.* 2006; STECF 2017). This 6% female participation rate cited in Salz *et al.* (2006) was applied to the estimated number of small-scale fishers in 2016. This number would be higher if it were assumed that women were mostly participating in the small-scale sub-sector; however, for the purposes of this study, and to be conservative, the 6% was used.

**Uncertainty:** This estimate receives an uncertainty score of 3 as the estimate was derived using a combination of sources that were not all from within the last decade and a recent report highlighted data quality issues for Greece (STECF 2017).

1. **Italy**

Employment in fishing activities in Italy was estimated at 25,787 in 2015, with approximately 49% of this being in the small-scale sub-sector (STECF 2017). Female participation in fishing was estimated at 8% in 2003 (Salz *et al.* 2006; STECF 2018).

**Estimate and Assumptions:** Participation by women in small-scale fishing activities is 8% out of a total of 12,635 small-scale fishers (Salz *et al.* 2006; STECF 2017). The female participation rate in fishing of 8% from 2003 was applied to the estimated number of small-scale fishers in 2015, assuming female participation rates did not change considerably over that period and assuming women involved directly in fishing activities participate mainly in the small-scale sub-sector.

**Uncertainty:** The estimate for fishing gets a score of 3 as the data were from more than a decade ago and likely only reflect permanent positions.

1. **Spain**

Nation-wide, Spain’s fisheries labour force includes approximately 92,800 individuals, with roughly 27% of these being women (Frangoudes *et al.* 2008a). Looking closer at the composition of this sector, there are 1200 women in fishing jobs, which overall represents 2% of the total number of fishers but would be higher if only considering the small-scale sector.

At the local level, women in Galicia have gained some recognition for their prominent role in the shellfish fishery as important actors throughout the shellfish value chain from harvesting to processing and marketing (Meltzoff 1995; Frangoudes *et al.* 2008b). Women are thought to represent approximately 90% of shellfish harvesters in Galicia, yet until very recently, their work was not formally recognized (Frangoudes *et al.* 2008a). Fisheries statistics for Galicia indicated that in 2015, out of a total of 8,460 (wild capture) shellfish harvesters, approximately were 469 women, while a much higher number of women were involved in the harvest of cultured shellfish (OCUPESCA 2017).

**Estimate and Assumptions:** Participation by women in small-scale fishing activities is 9% based on 1,200 out of 7,902 small-scale fishers (Goupement Monfort-Baelde-Vouhe 2017; STECF 2017). Assuming the participation rate of 2% cited in the literature was based on total fisheries employment, the rate of 9% was calculated based on the number of women cited in the literature related to the total participation in the small-scale sub-sector.

**Uncertainty:** This estimate received a score of 3 as the data were considered rigorous with several sources providing similar estimates.

**Western Europe**

1. **France**

Fishing in France continues to be an activity dominated by men; however, some women fish by foot and on boats, and many more women contribute to the fisheries sector in a wide range of fisheries-related activities, including processing, marketing, accounting, management, research, and as boat owners (Frangoudes and Keromnes 2008; Quist *et al.* 2010; Villemur and Angouillant 2015). Estimates of female participation by women in extractive activities suggest that women represent 3% of the participants in fishing activities (Frangoudes and Keromnes 2008; OECD 2015). Looking specifically at small-scale and coastal fishing, there are 204 women out of a total of 11,670 with an additional 117 women out of 1,227 who fish on foot. Women are much more prevalent in the harvest of cultured shellfish. However here, I focus only on capture fisheries. The above numbers on partially represent the labour contributions by women in fishing operations, especially the wives of fishers who are often heavily involved in fishing operations on shore and at sea—work that is often unpaid.

In 1998, France introduced legislation that allowed fishermen’s wives to voluntarily apply for *Collaborative Spouse Status*, which, in 2007 became mandatory (Frangoudes *et al.* 2008a). This provided legal status and benefits to fishermen’s wives, who had long been providing inputs to family-fishing businesses without support or recognition. Throughout this struggle for recognition, women also organized themselves into fisheries unions and organizations, separate from those representing men (MacAlister Elliott and Partners LTD 2002). These women’s organizations focused mainly on social issues, such as safety at sea and working conditions, while fisheries management and decision-making largely remained the domain of fishermen’s organizations (Quist *et al.* 2010).

**Estimate:** Participation by women in small-scale fishing activities is 2.5% out of a total of 12,897 small-scale, coastal and foot fishers (Goupement Monfort-Baelde-Vouhe 2017).

**Uncertainty:** Although the level of detail available for this country far exceeds that of other countries, the sources indicate a high degree of uncertainty (Goupement Monfort-Baelde-Vouhe 2017); therefore, this estimate for female participation in fishing receives a score of 2.

1. **Germany**

Women in Germany’s fisheries sector are more concentrated in fishing-related work than in the productive aspects of fishing, representing 1% of small-scale fisher (Salz *et al.* 2006).

**Estimate and Assumptions:** Participation by women in small-scale fishing activities is 1% out of a total of 724 people in small-scale fishing (Salz *et al.* 2006; STECF 2017). A female participation rate in fishing of 1% from 2003 was applied to the estimated number of small-scale fishers in 2015, assuming female participation rates did not change considerably over that time period and assuming women involved directly in fishing activities participate mostly in the small-scale sub-sector.

**Uncertainty:** This estimate received an uncertainty score of 3 as the data were from more than a decade ago and likely only reflect permanent positions.

1. **Netherlands**

In the Netherlands, women are thought to represent between 1-5% of fishers, while representing 39-43% of processing sector employment (MacAlister Elliott and Partners LTD 2002; Frangoudes *et al.* 2008a). However, these estimates overlook the many additional contributions by women to the fisheries-related economy through their shore-based work in running family-based fishing enterprises, in managing their households and in supporting the community while men are at sea fishing, often for an extended time period (Quist 2008; Quist *et al.* 2010). In the early 2000s, a network of fishermen’s wives came together to address various issues including sustainability in fisheries, community wellbeing, and better recognition of the role of women in the sector (Quist 2008). This women-in-fisheries network, known as *VinVis*, has been advocating, alongside other similar networks across Europe, for greater recognition and legal status for the unpaid labour contributions of women in the fishing industry (Frangoudes *et al.* 2008a). Despite some progress in gaining status and recognition, the role of women in fisheries is still absent in Dutch fishery statistics (Quist *et al.* 2010), reflecting a broader lack of recognition and enumeration of the informal, unpaid work done, in large part, by women, that is not captured by National accounts across all economic sectors, including fisheries.

**Estimate:** Participation by women in small-scale fishing activities is 5% out of a total of 1,200 coastal fishers in 2013 (MacAlister Elliott and Partners LTD 2002; OECD 2015).

**Uncertainty:** This estimate receives a score of 2 since the data used was from more than 10 years ago.

**Oceania**

**Australia & New Zealand**

1. **Australia**

The role of women in the Australian fishing industry and their contributions to fisheries output and productivity are poorly recognized (Aslin *et al.* 2000). With fisheries statistics for Australia being aggregated with other resource sectors and likely overlooking many of the informal work of women in running family fishing businesses (Shaw *et al.* 2015), the available data likely reflects only part of the picture. Fisheries Statistics presented by the Organization for Economic Development (OECD) provide employment numbers for Australia, disaggregated by sex for certain years with 2011 being the most recent estimate, indicating a female participation rate of approximately 12% for the fisheries sector (OECD 2015). Overall, the statistics collected and disseminated by the Government of Australia do not provide a clear picture of women in the fisheries sector, where they are involved, and in what numbers.

**Estimate and Assumptions:** Participation by women in coastal marine fishing activities is 12.8% out of a total of 5,050 people engaged in coastal harvest fisheries in 2013 (OECD 2015). The estimate is based on the annual average from the most recent three years (2009-2011) where the OECD employment data for Australia are disaggregated by sex for the category of Coastal Marine Fisheries. The total is based on a more recent estimate (2013), where the data have not been disaggregated by sex.

**Uncertainty:** The estimate for female participation in fishing receives an uncertainty score of 2. Although this estimate was based on national statistical data, it was likely not a comprehensive estimate, and it is unclear how these data were disaggregated from other sectors. Additionally, National statistics have been criticized for not properly reflecting women in the sector (Aslin *et al.* 2000).

1. **New Zealand**

In New Zealand’s fisheries sector, men dominate the capture sub-sector, while women are concentrated in the seafood-processing sector. However, women play an active role in fishing operations and businesses, with an increasing number of women working with men, often as husband and wife teams on boats as crew and skippers (Lambeth *et al.* 2014). Quantitative estimates for participation of this sort, disaggregated by sex, were not located.

**Estimate and Assumptions:** Female participation in small-scale fishing activities is 12.8% out of a total of 4,300 people in small-scale fishing activities (Teh and Sumaila 2013). In the absence of available estimates of female participation in fishing activities for New Zealand, a benefit transfer approach was used with the estimate derived from neighboring Australia.

**Uncertainty:** This estimate received a score of 1 since it was based on data for another country.

**Melanesia**

1. **Fiji**

Women in Fiji are involved in subsistence fishing and are increasingly becoming involved in the commercial fisheries sector (Lambeth *et al.* 2014). Women dominate the subsistence fishing sector, accounting for the majority of finfish catches for home consumption and representing almost half of small-scale fishers across Fiji (Kronen and Vunisea 2009; SPC 2013). Women are heavily involved in the mud crab fishery, which is an important income source for fishers in Bua Province (Thomas *et al.* 2018). More recently, women have become involved in the lucrative *bêche-de-mer* fishery as divers; however, a recent study found that women earn 47% less than men in this fishery (Purcell *et al.* 2018).

**Estimate and Assumptions:** Female participation in small-scale fishing activities is 46% out of a total of 12,000 small-scale fishers (SPC 2013; Gillett 2016). With fisher population estimates ranging from 12,000 to 40,000 people involved in artisanal and subsistence fishing either full or part-time, an estimate of 12,000 was used here to remain conservative.

**Uncertainty:** The estimate for fishing received a score of 2 as there was a considerable range in the cited number of the overall fisher population.

1. **Papua New Guinea**

Women’s role in fishing is much larger than is generally acknowledged with one study indicating that women catch at least 25% of the subsistence catch, which is mostly invertebrates from shallow inshore areas (Lambeth *et al.* 2014). While there is limited information on subsistence production for PNG, the catch of invertebrates (e.g., *bêche-de-mer*, trochus and other shellfish), for both commercial and subsistence purposes is considered to exceed the catch of finfish (Lambeth *et al.* 2014). Women also play an important role in fishing-related activities, dominating the processing of small-scale fisheries catches and being involved in the marketing of fish (Gillett 2016).

In terms of the number of participants in the fishing industry, no recent estimates exist (Gillett 2016). Data collected in the 1990s and early 2000s continue to be used as a best estimate of the small-scale fisher population, with some recent work being done to estimate the gender composition of small-scale fishers (SPC 2013).

**Estimate and Assumptions:** Female participation in small-scale fishing activities is 48% out of a total of 120,000 people involved in subsistence fishing activities (SPC 2013; Gillett 2016). Estimates of small-scale fisher population for PNG ranged from several thousand to almost half a million, but to be conservative, the estimate of 120,000 people involved in direct fishing activities was used.

**Uncertainty:** This estimate received a score of 2 since the data used was not that recent.

1. **Solomon Islands**

In the Solomon Islands, women make up a large proportion of the workforce in tuna and coastal fisheries supply chains; however, there is little or no data available to measure the extent of their engagement. In addition to processing activities, women also participate directly in fishing activities and as support for men’s fishing activities, including food, trade, and financial backup (Buga and Vuki 2012; Krushelnytska 2015). There is considerable variation between communities and from one province to another in terms of female participation in fishing activities where, in some communities, participation by women is high, while it is low in others (Kruijssen *et al.* 2015).

In 2004 there were an estimated 5,114 people formally employed in fishing (IMF, 2005). However, there are many more (including many women) who are not accounted for in this estimate because they were engaged informally in fishing and related activities. There are no comprehensive estimates of female participation in the fisheries sector or the ratio of men to women in the sector, which includes formal and informal sectors as main and secondary activities (Krushelnytska 2015). Overall in the Pacific Island region, women are responsible for over half of small-scale fisheries catches, contributing significantly to food and to livelihood security (Harper *et al.* 2013), with women in the Solomon Islands providing much of the seafood for home consumption (Pacific Community 2018).

**Estimate and Assumptions:** Female participation in small-scale fishing activities is 42% out of a total of 7,700 small-scale fishers (Teh and Sumaila 2013; Amos 2014). The female participation rate of 42% was applied to the total number of small-scale fishers estimated by Teh and Sumaila (2013), which was considered a conservative estimate as Gillett (2016) suggests as many as 175,000 people are likely involved in subsistence fishing activities when both men and women are included.

**Uncertainty:** This estimate receives a score of 2, as the estimate was derived by combining several data sources.

**Micronesia**

1. **Federal State of Micronesia**

In the Federated States of Micronesia, both men and women participate in inshore fishing activities for subsistence and artisanal purposes (Vali *et al.* 2014); However, there are clear distinctions in the gendered division of responsibilities, with women collecting crabs and other invertebrates that inhabit intertidal areas while men are responsible for catching fish and lobsters using spears or via free-diving and other boat-based fishing (Chapman 1987; Lambeth 2000).

There is considerable variation between states in the participation by women in fisheries, with higher involvement by women in Kosrae and Chuuk and lower in Pohnpei and Yap (Lambeth *et al.* 2002). There is also variation within states with, for example, women in the outer islands of Yap being more likely to collect from the reef and fish using hand lines than women from the main group of Yap islands (Lambeth *et al.* 2002). In Yap, an estimated 20% of fishers are women, while in Chuuk 32% are women (Gillett 2016).

Fisheries participation data for FSM was not readily available. Fisheries employment statistics for FSM indicate that approximately 250 people formally employed in the fishing industry (Gillett 2016). This likely reflects only industrial sector participation. Formal employment in fisheries is quite limited with the majority of those involved, including women, working informally in the small-scale sector (FAO 2010b).

**Estimate:** Female participation in small-scale fishing activities is 25% out of a total of 38,000 small-scale fishers (Teh and Sumaila 2013).

**Uncertainty:** This estimate received a score of 3 as there were relatively robust data with some agreement among sources.

1. **Kiribati**

In Kiribati, women have a significant role in fisheries, both in the subsistence and cash sectors (Taniera and Mitchell 1995). Women are involved in both fishing and in processing, making important contributions to food security and to household income (Fay *et al.* 2007). Fishing activities are mainly done by foot, gleaning the shore and reefs for invertebrates; however, some women also fish the outer islands from boats using gill nets, rods and lines, and traditional fish traps (Taniera and Mitchell 1995).

**Estimate:** Female participation in small-scale fishing activities is 35% out of a total of 23,000 small-scale fishers (Teh and Sumaila 2013; Amos 2014).

**Uncertainty:** The estimate for female participation in fishing activities receives a score of 3.

1. **Palau**

Women in Palau have always played an important role in the fisheries economy through their reef-gleaning activities (Lambeth *et al.* 2014). Women are particularly involved in the sea cucumber fishery, with most of the collection and processing activities being done by women (Pakoa *et al.* 2014). Palauan are also involved in other fisheries activities, including marketing of seafood and, more recently, some women also go out fishing in small boats (Lambeth 1999). The objective of fishing for women is often subsistence while men focus more on catching fish for commercial purposes (Fairbairn-Dunlop 2014).

Fisheries employment statistics for Palau estimate approximately 460 people in the primary sector; however, this estimate likely only accounts for formal employment and misses the many people, including women, involved informally in fishing and related activities. Added to this are at least another 933 subsistence fishers (Gillett 2016), which brings the estimate of fishers in 2008 to approximately 1400. An estimated 32% of small-scale fishers are women (SPC 2013).

**Estimate:** Female participation in small-scale fishing activities is 32% out of a total of 1,400 small-scale fishers (SPC 2013; Gillett 2016).

**Uncertainty:** This estimate receives a score of 3 as the data were relatively robust.

**Polynesia**

1. **French Polynesia**

In French Polynesia, men dominate finfish fisheries while women participate more prominently in invertebrate fisheries (Kronen *et al.* 2008). Women typically fish close to shore, targeting the sheltered coastal reef habitats. While statistics on the number of people participating in fishing and fishing-related activities in French Polynesia were limited, the number of people employed in fisheries is estimated around 10,500 (Gillett 2016), with an estimated fisher population of almost 4,000 in Moorea alone (Yonger 2002). In terms of female participation in fishing, an estimated 22% of small-scale fishers are women (SPC 2013).

**Estimate and Assumptions:** Female participation in small-scale fishing activities is 22% out of a total of 10,500 small-scale fishers (SPC 2013; Gillett 2016). The details provided in Gillett (2016) for total fisheries employment were taken to represent participation in small-scale fishing activities in French Polynesia.

**Uncertainty:** The estimate for participation in fishing receives a score of 3.

1. **Samoa**

Women in Samoa participate in subsistence fishing activities, such as gleaning along the shoreline, lagoon, and reefs at low tide. Traditionally women fish inshore with basic fishing gear, targeting invertebrates and small finfish. Women make up between 17% and 22% of fishers in Samoa and catch roughly 23% of the total weight of seafood (Amos 2014; Lambeth *et al.* 2014). Fishers, both male and female targeting finfish, are mainly for the commercial market. While fishers of all genders target coastal reef and lagoon habitats, only men fish for pelagic species, in the open oceans and in mangroves. However, a few women fish the outer reefs. In terms of gendered division of labour in target species and habitats for invertebrate fisheries, women mainly target soft bottom species, whereas men collect clams, octopus, lobster, and *bêche-de-mer*, either gleaning or diving along reef tops and in mangrove areas (Gillett 2016). Of Total annual catch taken by women, including both finfish and invertebrates is estimated by (Tiitii *et al.* 2014) to be almost 3,000 t·year^-1^.

**Estimate:** Female participation in small-scale fishing activities is 20% out of a total of 12,000 small-scale fishers (Teh and Sumaila 2013; Amos 2014; Gillett 2016).

**Uncertainty:** This estimate receives a score of 3 as several, reliable sources indicated similar estimates.

1. **Tonga**

In Tonga, both women and men exploit reef and lagoon resources, but they do so in different ways with women and children gathering seaweed and invertebrates by hand and using simple tools, such as spears and traps, whereas men fish use spears, hooks, nets, and traps (Malm 2009). Tonga differs somewhat from the rest of the Pacific Island countries in that women only participate in the collection of invertebrates, while men participate in both finfishing and invertebrate collection (Fairbairn-Dunlop 2014). Children play a significant role in fisheries, with both girls and boys involved from an early age in the harvest of marine resources, mainly for home consumption, but also for income generation (Kronen 2004). Sea cucumbers, collected by women from intertidal and shallow subtidal areas, are an increasing source of income for women in Tonga (Purcell *et al.* 2016).

Women collect substantial volume of seafood through their reeftop gleaning activities every year with average annual catch rates, for example in Lofanga, of approximately 600 kg fisher^-1^ year^-1^ (Kronen and Malimali 2009). The activities of Lofongan fisherwomen contribute to the high per capita consumption of invertebrates and to household income through their fishing, processing, and marketing octopus and giant clams (Kronen and Malimali 2009).

**Estimate and Assumptions:** Female participation in small-scale fishing activities is 17% out of a total of 4,800 small-scale fishers (Teh and Sumaila 2013; Gillett 2016). With estimates of the fisher population of Tonga ranging from 1,000 to over 10,000 a female participation rate of 17% was applied to the small-scale fisher population of 4,800 from Teh and Sumaila (2013).

**Uncertainty:** This estimate receives a score of 3 as several sources indicated similar numbers.

### S3 Appendix References

Alaedini, P. and Razavi, M.R. (2005) Women’s Participation and Employment in Iran: A Critical Examination. *Critique: Critical Middle Eastern Studies* **14**, 57–73.

Alonso-Población, E. and Siar, S. V. (2018) Women’s participation and leadership in fisherfolk organizations and collective action in fisheries. Rome.

Amos, M. (2014) Growing and empowering women in fisheries: Work in the Pacific region. *SPC Women in Fisheries Information Bulletin* **25**, 3–5.

Anna, Z. (2012) The Role of Fisherwomen in the Face of Fishing Uncertainties on the North Coast of Java, Indonesia. *Asian Fisheries Science* **25S**, 145–158.

Araneda, D., Salas, J., Pinto, A. and Alvarez, M. (2005) Questioning invisibility. *Yemaya* **19**, 6–7.

ArtFiMed (2009) Diagnostique initial des sites de pêche artisanale du Maroc et de Tunisie. Malaga.

Ashaletha, S., Ramachandran, C., Immanuel, S., Diwanj, A.D. and Sathiadhas, R. (1995) Changing Roles of Fisherwomen of India: Issues & Perspectives. Kochi.

Asian Development Bank (2011) The Informal Sector and Informal Employment in Indonesia. Asian Development Bank, Mandaluyong City.

Aslin, H.J., Webb, T. and Fisher, M. (2000) Fishing for women: understanding women’s roles in the fishing industry. Canberra.

Bangladesh Bureau of Statistics. (2015) Labour Force Survey 2013. Available at: http://203.112.218.65:8008/WebTestApplication/userfiles/Image/LatestReports/LabourForceSurvey.2013.pdf [Accessed February 2, 2018].

Barnes-Mauthe, M., Oleson, K.L.L. and Zafindrasilivonona, B. (2013) The total economic value of small-scale fisheries with a characterization of post-landing trends: An application in Madagascar with global relevance. *Fisheries Research* **147**, 175–185.

Barnes, D.K.A. and Rawlinson, K.A. (2009) Traditional coastal invertebrate fisheries in south-western Madagascar. *Journal of the Marine Biological Association of the United Kingdom* **89**, 1589–1596.

Belhabib, D., Divovich, E. and Pauly, D. (2016a) Angola. In: *Global Atlas of Marine Fisheries: A Critical Appraisal of Catches and Ecosystem Impacts*. (eds D. Pauly and D. Zeller). Island Press, Washington, DC, p 187.

Belhabib, D., Koutob, V., Sall, A., Lam, V.W.Y. and Pauly, D. (2014) Fisheries catch misreporting and its implications: The case of Senegal. *Fisheries Research* **151**, 1–11.

Belhabib, D., Pauly, D., Harper, S. and Zeller., D. (2016b) Algeria. In: *Global Atlas of Marine Fisheries: A Critical Appraisal of Catches and Ecosystem Impacts*. (eds D. Pauly and D. Zeller). Island Press, Washington, DC, p 186.

Belhabib, D., Sumaila, U.R. and Pauly, D. (2015) Feeding the poor: Contribution of West African fisheries toemployment and food security. *Ocean and Coastal Management* **111**, 72–81.

Berazaluce Maturana, P., Burgos González, J. and Bordas Coddou, A. (2017) Mujeres y hombres en el secto Pesquero y Acuicultor de Chile 2017. Santiago.

Bonfiglioli, A. and Hariri, K.I. (2004) Small-scale Fisheries in Yemen: Social Assessment and Development Prospects. Washington, DC.

Branch, G.M., May, J., Roberts, B., Russell, E. and Clark, B.M. (2002) Case studies on the socio-economic characteristics and lifestyles of subsistence and informal fishers in South Africa. *South African Journal of Marine Sciences* **24**, 439–467.

Brugère, C. and Maal, B. (2014) Study of fisheries and aquaculture value chains in Mozambique. Oslo.

Brummett, R., Youaleu, J., Tiani, A.-M. and Kenmegne, M. (2010) Women’s traditional fishery and alternative aquatic resource livelihood strategies in the Southern Cameroonian Rainforest. *Fisheries Management and Ecology* **17**, 221–230.

Buga, B. and Vuki, V. (2012) The people of the artificial island of Foueda, Lau Lagoon, Malaita, Solomon Islands: Traditional fishing methods, fisheries management and the roles of men and women in fishing. *SPC Women in Fisheries Information Bulletin*, 42–44.

Bultel, E., Doherty, B., Herman, A., Le Manach, F. and Zeller, D. (2015) An update of the reconstructed marine fisheries catches of Tanzania with taxonomic breakdown. In: *Fisheries catch reconstructions in the Western Indian Ocean, 1950–2010.*, Vol. 23 (2). (eds F. Le Manach and D. Pauly). The University of British Columbia, Vancouver, pp 151–161.

Calhoun, S., Conway, F. and Russell, S. (2016) Acknowledging the voice of women: implications for fisheries management and policy. *Marine Policy* **74**, 292–299.

Carney, J. (2017) “The mangrove preserves life”: Habitat of African survival in the Atlantic world. *Geographical Review* **107**, 433–451.

Central Marine Fisheries Research Institute (2010) Marine Fisheries Census 2010. New Delhi.

Chapman, M.D. (1987) Women’s fishing in Oceania. *Human Ecology* **15**, 267–288.

Di Ciommo, R.C. (2007) Pescadoras e pescadores: a questão da equidade de gênero em uma reserva extrativista marinha. *Ambiente & sociedade* **10**, 151–163.

Cliffe, P. and Akinrotimi, O. (2015) Role of Women in Fishery Activities in some coastal communities of Rivers State, Nigeria. *International Journal of Agricultural Research* **10**, 24–32.

de la Torre-Castro, M. De, Fröcklin, S., Börjesson, S., Okupnik, J. and Jiddawi, N.S. (2017) Gender analysis for better coastal management – Increasing our understanding of social-ecological seascapes. *Marine Policy* **83**, 62–74.

Delaney, A. and Yagi, N. (2017) Implementing the Small-Scale Fisheries Guidelines: Lessons from Japan. In: *The Small-Scale Fisheries Guidelines*. (eds S. Jentoft, R. Chuenpagdee, M. Barragán-Paladines and N. Franz), MARE Publi. Springer, Cham, Switzerland.

Delgado-Gustavson, V. (2011) Fishing Communities: Gender, Economic Life, and Welfare Regimes. Master’s Thesis. Universitas Bergensis.

Deme, M., Thiao, D., Fambaye, N.S., Sarre, A. and Diadhiou, H.D. (2012) Dynamique des Populations de Sardinelles en Afrique du Nord-Ouest: Contraintes Environnementales, Biologiques et Socio Economiques. Narragansett, RI.

Diegues, A.C. (2008) Marine protected areas and artisanal fisheries in Brazil. Chennai.

Döring, R., Carvalho, N. and Virtanen, J. (2012) Scientific, Technical and Economic Committee for Fisheries (STECF) Economic Performance of the EU Fish Processing Industry Sector. Luxembourg.

Durai, J.A. and Dhanalakshmi, J. (2015) Role of women in fishery sector in Tamil Nadu. *International Journal of Application or Innovatioin in Engineering and Management* **4**, 9–13.

Failler, P., Beyens, Y. and Asiedu, B. (2014) Value chain analysis of the fishery sector in Ghana with focus on quality, environmental, social, sustainable, food safety, organic requirements and its compliance infrastructure. Accra.

Fairbairn-Dunlop, T.P. (2014) A Pacific Way of Counting. In: *Counting on Marilyn Waring: New Advances in Feminist Economics*. (eds M. Bjornholt and A. McKay). Demeter Press, Bradford, pp 119–133.

FAO (2014) Bangladesh. Available at: http://www.fao.org/fishery/facp/BGD/en [Accessed February 2, 2018].

FAO (2010a) Brazil. Available at: http://www.fao.org/fishery/facp/BRA/en [Accessed May 1, 2018].

FAO (2007a) Cameroon. Available at: http://www.fao.org/fishery/docs/DOCUMENT/fcp/fr/FI_CP_CM.pdf [Accessed December 19, 2017].

FAO (2015) Cuba. Available at: http://www.fao.org/fishery/facp/CUB/es [Accessed April 16, 2018].

FAO (2008a) Dominican Republic. Available at: http://www.fao.org/fishery/facp/DOM/en [Accessed April 16, 2018].

FAO (2017) FAO project supports women clam collectors in Tunisia. Available at: http://www.fao.org/in-action/women-in-agrifood-value-chains/fao-project-supports-women-clam-collectors-in-tunisia/en/ [Accessed December 13, 2017].

FAO (2007b) Gabon. Available at: http://www.fao.org/fishery/docs/DOCUMENT/fcp/fr/FI_CP_GA.pdf [Accessed December 19, 2017].

FAO (2016) Jamaica. Available at: http://www.fao.org/fishery/facp/JAM/en [Accessed April 16, 2018].

FAO (2005a) Le Royaume du Maroc. Available at: http://www.fao.org/fishery/docs/DOCUMENT/fcp/fr/FI_CP_MA.pdf [Accessed December 18, 2017].

FAO (2005b) Libya. Available at: http://www.fao.org/fishery/docs/DOCUMENT/fcp/en/FI_CP_LY.pdf [Accessed December 18, 2017].

FAO (2009a) Malaysia. Available at: http://www.fao.org/fishery/facp/MYS/en [Accessed February 12, 2018].

FAO (2008b) Mozambique. Available at: http://www.fao.org/fishery/docs/DOCUMENT/fcp/en/FI_CP_MZ.pdf [Accessed January 9, 2018].

FAO (2007c) Namibia. Available at: http://www.fao.org/fishery/facp/NAM/en [Accessed January 12, 2018].

FAO (2007d) Nigeria. Available at: http://www.fao.org/fishery/facp/NGA/en [Accessed December 13, 2017].

FAO (2010b) The Federated States of Micronesia. Available at: http://www.fao.org/fishery/facp/FSM/en [Accessed July 5, 2018].

FAO (2009b) The Kingdom of Thailand. Available at: http://www.fao.org/fishery/facp/THA/en [Accessed January 31, 2018].

FAO (2005c) The Republic of El Salvador. Available at: http://www.fao.org/fishery/docs/DOCUMENT/fcp/es/FI_CP_SV.pdf [Accessed April 15, 2018].

FAO (1994) The role of women in Agriculture. Available at: http://www.fao.org/docrep/V8195E/v8195e01.htm#TopOfPage [Accessed December 18, 2017].

FAO (2013) The Sultanate of Oman. Available at: http://www.fao.org/fishery/facp/OMN/en [Accessed February 1, 2018].

FAO (2005d) Tunisia. Available at: http://www.fao.org/fishery/facp/TUN/fr [Accessed December 13, 2017].

Fay, L., Vuki, V., Sauni, S. and Tebano, T. (2007) Anadara fishing supports urban households in Tarawa, Kiribati and Suva, Fiji. *SPC Women in Fisheries Information Buletin* **17**, 19–26.

Ferrari, B. (2016) Fighting invisibility: Fisherwomen in Brazil demand to be heard on their right to social security and decent work. *Yemaya* **52**, 4–5.

Fisheries and Fisheries Administration of the Ministry of Agriculture (2014) *China Fisheries Yearbooks, 2008-2014*. China Fishery Yearbook Publishing House.

Fishery Survey of India (2012) Marine Fisheries Census 2010: Union Territories of Andaman & Nicobar and Lakshadweep Islands. New Delhi.

Fitriana, R.I.A. and Stacey, N. (2012) The Role of Women in the Fishery Sector of Pantar Island, Indonesia. *Asian Fisheries Science Special Issue* **25S**, 159–175.

Ford, J.D. and Goldhar, C. (2012) Climate change vulnerability and adaptation in resource dependent communities: A case study from West Greenland. *Climate Research* **54**, 181–196.

Frangoudes, K., Carrol, M., Holmyard, N., Marcianiak, B., Cristina, M., Pascual-Fernandez, J., Marugan Pintos, B., Ronn, C. and Quist, C. (2008a) The role of women in the sustainable development of European Fisheries Areas. Brussels.

Frangoudes, K. and Keromnes, E. (2008) Women in Artisanal Fisheries in Brittany, France. *Development* **51**, 265–270.

Frangoudes, K., Marugán-Pintos, B. and Pascual-Fernández, J.J. (2008b) From open access to co-governance and conservation: The case of women shellfish collectors in Galicia (Spain). *Marine Policy* **32**, 223–232.

Fröcklin, S., de la Torre-Castro, M., Håkansson, E., Carlsson, A., Magnusson, M. and Jiddawi, N.S. (2014) Towards improved management of tropical invertebrate fisheries: including time series and gender. *PloS one* **9**, 1–12.

Fröcklin, S., de la Torre-Castro, M., Lindström, L. and Jiddawi, N.S. (2013) Fish traders as key actors in fisheries: gender and adaptive management. *Ambio* **42**, 951–62.

Gammage, S. (1996) The tattered net of statistics. *SAMUDRA Report* **16**, 36–40.

Garcia, A. (2000) Perú: Gender Issues in the Fisheries Sector. In: *Workshop on Gender and Coastal Fishing Communities in Latin America, 10 to 15 June 2000, Prainha do Canto Verde, Ceara, Brazil*. ICSF, Chennai, pp 97–110.

Gerrard, S. (2005) Research Relations and Globalization: Feminist Reflections on the Informant-Researcher Relationship. In: *Changing Tides: Gender, Fisheries and Globalization*. (eds B. Neis, M. Binkley, S. Gerrard and M.C. Maneschy). Fernwood Publishing, Halifax, pp 215–228.

Gerrard, S. (2018) Then and Now—Women in Norway’s Fisheries. *Yemaya* **56**, 7–10.

Gerrard, S. (2006) Women, men and fishing quotas. *Yemaya* **22**, 1–2.

Gervásio, H.F. (2014) Governing the intertidal subsistence fisheries in Mozambique: Vulnerability, marginalization and policy mismatches Case study of the district of Palma (The Province of Cabo Delgado).

Gillett, R. (2016) *Fisheries in the Economies of Pacific Island Countries and Territories*. Pacific Community, Noumea.

Glaeser, B. and Glaser, M. (2011) People, fish and coral reefs in Indonesia: A contribution to social-ecological research. *Gaia* **20**, 139–141.

Göncüoǧlu, H. and Ünal, V. (2011) Fisherwomen in the Turkish fishery, southern Aegean Sea. *Journal of Applied Ichthyology* **27**, 1013–1018.

Göncüoğlu, H., Ünal, V. and Kızılkaya, Z. (2015) Supporting Fisherwomen in Small-Scale Fisheries in Turkey. In: *First Regional Symposium on Sustainable Small-Scale Fisheries in the Mediterranean and Black Sea*. (eds A. Srour, N. Ferri, D. Bourdenet, D. Fezzardi and A. Nastasi). Rome, pp 447–451.

Gopal, N., Sruthi, P., Jayalal, L., Meenakumari, B., Rajaratnam, S. and McDougall, C. (2017) Gender Baselines in Fisheries and Aquaculture Value Chain in India: A Systematic Review. In: *Gender in Aquaculture and Fisheries*. Asian Fisheries Society, Kerala.

Goupement Monfort-Baelde-Vouhe (2017) La place des femmes dans les secteurs pêche et aquaculture en France. Paris.

Grandcolas, D. (1997) Les femmes et la collecte des huitres dans le Saloum (Senegal). Dakar.

Grant, S.C. (2004) Caribbean women in fishing economies. In: *Proceedings of the Fifty Fifth Annual Gulf and Caribbean Fisheries Institute Conference*. pp 68–77.

Guard, M. and Mgaya, Y.D. (2002) The Artisanal Fishery for Octopus cyanea Gray in Tanzania. *Ambio* **31**, 528–536.

Gueye, G. (2016) Voices from African Artisanal Fisheries. Stockholm.

Harper, S., Grubb, C., Stiles, M. and Sumaila, U.R. (2017) Contributions by Women to Fisheries Economies: Insights from Five Maritime Countries. *Coastal Management* **45**, 91–106.

Harper, S., Zeller, D., Hauzer, M., Pauly, D. and Sumaila, U.R. (2013) Women and fisheries: contribution to food security and local economies. *Marine Policy* **39**, 56–63.

Herrera, A., Betancourt, L., Silva, M., Lamelas, P. and Melo, A. (2011) Coastal fisheries of the Dominican Republic. In: *Coastal fisheries of Latin America and the Caribbean*. (ed A.C. and J.C.S. Salas, R. Chuenpagdee). FAO, Rome, pp 175–217.

Howell, L.A. (2002) Perspectives on women in fisheries in North America. In: *Global Symposium on Women in Fisheries: Sixth Asian Fisheries Forum*. (eds M.J. Williams, N.H. Chao, P.S. Choo, Matics, K., Nandeesha, M.C., Shariff, M., Siason, I., Tech, E. and Wong, J.M.C.). ICLARM-The World Fish Center, Kaohsiung, pp 183–188.

Immanuel, S., Pillai, V.N., Vivekanandan, E., Kurup, K.N. and Srinath, M. (2003) A Preliminary Assessment of the Coastal Fishery Resources in India - Socioeconomic and Bioeconomic Perspective. Penang.

Immanuel, S. and Rao, G.S. (2009) The Status of Fisherwomen in Andhra Pradesh. *Indian Journal of Gender Studies* **16**, 411–423.

INEGI (2011) Pesca y acuicultura: Censos Económicos 2009. Aguascalientes.

INFOPESCA (2018) Red Latinoamericana de las Mujeres del Sector Pesquero [Latin American Network of Women working in the fisheries sector]. Available at: http://www.mujeres.infopesca.org/ [Accessed April 16, 2018].

Instituto Nacional de Estadística e Informática (2012) Perú - I Censo Nacional de la Pesca Artesanal del Ámbito Marítimo 2012 [Peru- First National Census of Artisanal Fishers]. Lima.

Jacquet, J., Fox, H., Motta, H., Ngusaru, A. and Zeller, D. (2010) Few data but many fish: Marine small-scale fisheries catches for Mozambique and Tanzania. *African Journal of Marine Science* **32**, 197–206.

Jiddawi, N.S. and Öhman, M.C. (2002) Marine Fisheries in Tanzania. *AMBIO: A Journal of the Human Environment* **31**, 518–527.

Johnstone, R. (2003) A More Central Role. *Yemaya* **14**, 7–8.

Kafarowski, J. (2006) Valuing local knowledge in the Canadian Arctic: How the involvement of local peoples results in relevant resource management decisions. In: *Global Symposium on Gender and Fisheries*. (eds P.S. Choo, S.J. Hall and M.J. Williams). WorldFish Center and Asian Fisheries Society, Penang, Malaysia, pp 169–173.

Koshy, N. and Sharma, C. (2007) Shoring Up. *SAMUDRA Report* **48**, 17–21.

Kronen, M. (2004) Alu toutai - Na laki qoli - Fun or duty: School children’s involvement in subsistence fisheries in Tonga and Fiji. *SPC Women in Fisheries Information Buletin* **14**, 9–17.

Kronen, M., Friedman, K., Pinca, S., Chapman, Lindsay Awiva, R., Pakoa, K., Vigliola, L., Boblin, P. and Magron, F. (2008) Pacific Regional Oceanic and Coastal Fisheries Development Programme French Polynesia Country Report. Noumea.

Kronen, M. and Malimali, S. (2009) The octopus fishery on Lofanga, Kingdom of Tonga. *SPC Women in Fisheries Information Bulletin* **19**, 11–16.

Kronen, M. and Vunisea, A. (2009) Fishing impact and food security – Gender differences in finfisheries across Pacific Island countries and cultural groups. *SPC Women in Fisheries Information Buletin*, 3–10.

Kruijssen, F., Albert, J., Morgan, M., Boso, D., Siota, F., Sibiti, S. and Schwarz, A.-M. (2015) Livelihoods, markets, and gender roles in Solomon Islands: Case studies from Western and Isabel Provinces. *SPC Women in Fisheries Information Bulletin* **26**, 24–36.

Krushelnytska, O. (2015) Toward Gender-Equitable Fisheries Management in Solomon Islands. Washington, DC.

Lambeth, L. (2000) An Assessment of the Role of Women in Fisheries in Pohnpei, Federated States of Micronesia. Noumea.

Lambeth, L. (1999) An Assessment of the Role of Women within Fishing Communities in the Republic of Palau. Noumea.

Lambeth, L., Hanchard, B., Aslin, H., Fay-Sauni, L., Tuara, P., Des Rochers, K., Vunisea Source, A. and Wong, J. (2014) An overview of the involvement of women in fisheries activities in Oceania. *SPC Women in Fisheries Information Bulletin* **25**, 21–33.

Lambeth, L., Hanchard, B., Aslin, H., Fay-Sauni, L., Tuara, P., Rochers, K.D. and Vunisea, A. (2002) An overview of the involvement of women in fisheries activities in Oceania. Noumea.

Laukitis, E. (2017) *Ocean notes: A book of seafaring women*. Salmon Sisters, Bristol Bay.

Lentisco, A. and Phuong Thao, H.T. (2013) Strengthening livelihoods: A Vietnamese fisheries programme helps. *SPC Women in Fisheries Information Bulletin* **23**, 45.

Libanova, E., Makarova, O., Gerasymenko, G., Aksyonova, S., Maidanik, I., Tkachenko, L., Lysa, O., Reut, A. and Otkydach, M. (2012) Analytical Research on Women’s Participation in the Labour Force in Ukraine. Kyiv.

Lim, C.P., Ito, Y. and Matsuda, Y. (2012) Braving the Sea: The Amasan (Women Divers) of the Yahataura Fishing Community, Iki Island, Nagasaki Prefecture, Japan. *Asian Fisheries Science* **25S**, 29–45.

MacAlister Elliott and Partners LTD (2002) Summary of the report “The Role of Women in Fisheries.” Luxembourg.

Mackenzie, C.L. (2001) The Fisheries for mangrove cockles, Anadara spp., from Mexico to Peru, with descriptions of their habitats and biology, the fishermen’s lives, and the effects of shrimp farming. *Marine Fisheries Review* **63**, 1–39.

Malm, T. (2009) Women of the coral gardens: The significance of marine gathering in Tonga. *SPC Traditional Marine Resource Management and Knowledge Information Bulletin* **25**, 2–15.

Máñez, K.S. and Pauwelussen, A. (2016) Fish Is Women’s Business Too: Looking at Marine Resource Use Through a Gender Lens. In: *Perspectives on Oceans Past*. (eds K.S. Máñez and B. Poulsen). Springer, Dordrecht, pp 193–211.

Marshall, N.T., Milledge, S.A.H. and Afonso, P.S. (1999) Stormy seas for marine invertebrates: trade in sea cucumbers, sea shells and lobsters in Kenya, Tanzania and Mozambique. Nairobi.

Matthews, E., Bechtel, J., Britton, E., Morrison, K. and McClennen, C. (2012) A Gender Perspective on Securing Livelihoods and Nutrition in Fish-dependent Coastal Communities. Bronx, NY.

Meltzoff, S.K. (1995) Marisquadoras of the Shellfish Revolution: The Rise of Women in Co-management on Illa de Arousa. *Journal of Political Ecology* **2**, 20–38.

Ministry of Agriculture Forestry and Fisheries (2016) The 90th Statistical Yearbook of Ministry of Agriculture Forestry and Fisheries Japan 2014/2015. Tokyo.

Monfort, M.C. (2015) The role of women in the seafood industry. GLOBEFISH Research Programme, Rome.

Munk-Madsen, E. (1998) The Norwegian fishing quota system: Another patriarchal construction? *Society & Natural Resources* **11**, 229–240.

National Statistical Office (2001) The 2000 Intercensal Survey of Marine Fishery. Bangkok.

Neis, B., Gerrard, S. and Power, N.G. (2013) Women and Children First: the Gendered and Generational Social- ecology of Smaller-scale Fisheries in Newfoundland and Labrador and Northern Norway. *Ecology and Society* **18**, 64.

Ngo Som, J. (1995) Women’s role in Cameroon fishing communities: the cases of Limbe and Kribi. IDAF Programme Report: Report of the working group on women’s key role and issues related to gender in fishing communities. Rome.

Nwabeze, G., Ifejika, P., Tafida, A., Ayanda, J., Erie, A. and Belonwu, N. (2013) Gender and Fisheries of Lake Nainji, Nigeria: A review. *Journal of Fisheries and Aquatic Sciences* **8**, 9–13.

OCUPESCA (2017) Enquisa sobre a poboación ocupada nos sectores da pesca e da acuicultura mariña en Galicia. Santiago de Compostela.

OECD (2015) OECD Review of Fisheries: Country Statistics 2014. OECD Publishing. Geneva.

Ogden, L.E. (2017) Fisherwomen-The uncounted dimension in fisheries management. *BioScience* **67**, 111–117.

Okeowo, T., Bolarinwa, J. and Ibrahim, D. (2015) Socioeconomic Analysis of Artisanal Fishing and Dominant Fish Species in Lagoon Waters of EPE and Badagry Areas of Lagos State. *International Journal of Research in Agriculture and Forestry* **2**, 38–45.

OSPESCA (2012) Encuesta Estructural de la pesca artesanal y la Acuicultura en Centroamérica: 2009-2011. Antiguo Cuscatlán.

Overå, R. (1992) Fish Mammies. The Role of Women in the Artisanal Fisheries Sector of Ghana. Master’s Thesis. University of Bergen.

Pacific Community (2018) Gender analysis of the fisheries sector - Solomon Islands. Noumea.

Pakoa, K., Simpson, R., Demei, L., Olsudong, Downette Salong, C., Rechelluul, P. and Fisk, D. (2014) The status of sea cucumber fisheries resources and management for Palau. Noumea.

Poonnachit-Korsieporn, A. (2000) Coastal fishing communities in Thailand. Bangkok.

Porter, M., Mwaipopo, R., Faustine, R. and Mzuma, M. (2008) Globalization and Women in Coastal Communities in Tanzania. *Development* **51**, 193–198.

Purcell, S.W., Lalavanua, W., Cullis, B.R., Cocks, N. and Purcell, S.W. (2018) Small-scale fishing income and fuel consumption: Fiji’s artisanal sea cucumber fishery. *ICES Journal of Marine Science* **75**, 1758–1767.

Purcell, S.W., Ngaluafe, P., Aram, K.T. and Lalavanua, W. (2016) Variation in postharvest processing of sea cucumbers by fishers and commercial processors among three Pacific Island countries. *SPC Bêche-de-mer Information Bulletin* **36**, 58–66.

Quist, C. (2008) VinVis: The women in fisheries network. *Yemaya* **27**, 5–7.

Quist, C., Frangoudes, K. and O’Riordan, B. (2010) ICSF-AKTEA WIF 2010 Strengthening the voice of women of fishing communities in Europe. Brest.

Raab, D. and Roche, D. (2005) A preliminary assessment of the artisanal fishery in the town of Pedro González, Archipelago of Las Perlas, Panama.

Rabbanee, F.K. and Yasmin, S. (2011) Role of Women in Processing and Marketing of Dry Fish from Coastal Bangladesh – An Exploratory Study. *East West Journal of Business and Social Studies* **2**, 39–62.

Raemaekers, S. and Sunde, J. (2015) Women in fisheries in Africa. *Yemaya* **50**, 10–11.

Al Rashdi, K.M. and McLean, E. (2014) Contribution of Small-Scale Fisheries to the Livelihoods of Omani Women: A Case Study of the Al Wusta Governorate. *Asian Fisheries Science* **27S**, 135–149.

Reedy-Maschner, K. (2009) Chercher Les Poissons: Gender Roles in an Aleut Indigenous Commercial Economy. In: *Gender, Culture and Northern Fisheries*. (ed J. Kafarowski). CCI Press, Edmonton, pp 3–28.

Reynolds, J., Abukhader, A. and Abdallah, A. (1995) The marine wealth sector of Libya: A development planning overview. Tripoli/Rome.

Rocha, L.M. (2013) “Ecologia Humana Manejo Participativo Da Pesca Do Búzio Anomalocardia brasiliana (Gmelin, 1791)(Bivalvia: Veneridae) Na Reserva De Desenvolvimento Sustentável Estadual Ponta Do Turbarão (RN).” Master’s Thesis. Universidade Federal Do Rio Grande Do Norte.

Rocha, L.M. and Pinkerton, E. (2015) Comanagement of clams in Brazil: a framework to advance comparison. *Ecology and Society* **20**, 7.

Salazar, H. and Castañeda, I. (2002) Background paper: Mexico-Women in Fisheries. In: *Workshop on Gender and Coastal Fishing Communities in Latin America: 10 to 15 June 2000, Prainha do Canto Verde, Ceara, Brazil*. International Collective in Support of Fishworkers, Chennai, pp 45–96.

Salz, P., Buisman, E., Smit, J. and de Vos, B. (2006) Employment in the fisheries sector: current situation (FISH/2004/4). Brussels.

Sasu, L. (1999) Breaking through culture. *Yemaya* **1**, 4–5.

Seilert, H. and Sangchan, S. (2001) Small-scale fishery in Southeast Asia: A case-study in Southern Thailand. Bangkok.

Shaw, J., Stocker, L. and Noble, L. (2015) Climate change and social impacts: women’s perspectives from a fishing community in Western Australia. *Australian Journal of Maritime & Ocean Affairs* **7**, 38–51.

Siason, I.M., Tech, E., Matics, K.I., Choo, P.S., Shariff, M., Heruwati, E.S., Susilowati, T., Miki, N., Shelly, A.B., Rajabharshi, K.G., Ranjit, R., Siriwardena, P.P.G.N., Nandeesha, M.C., Sunderarajan, M. (2002) Women In Fisheries in Asia. In: *Global Synposium on Women in Fisheries*. (eds Williams, M.J. Chao, N.H., Matics, K.I., Nandeesha, M.C., Shariff, M., Siason, I., Tech, E. and Wong, J.M.C.). The World Fish Center, Penang, Malaysia, pp 21–48.

Silva, C.N. (2000) Perú: Women in the Fisheries Sector. In: *Workshop on Gender and Coastal Fishing Communities in Latin America*. ICSF, Chennai, pp 111–113.

Silva, P. (2006) Exploring the Linkages between Poverty, Marine Protected Area Management, and the Use of Destructive Fishing Gear in Tanzania. New York.

Sloan, L., Kafarowski, J., Heilmann, A., Karlsdóttir, A., Udén, M., Angell, E. and Erlandsen, M.M. (2002) Women’s participation in decision-making processes in Arctic fisheries resource management, Arctic Council 2002-2004. Norfold.

Soumare, A. (2006) Senegal Role of Women in a Model of Community Management of Fish Resources and Marine Environments, Cayar. Dakar.

SPC (2013) Status report: Pacific Islands reef and nearshore fisheries and aquaculture 2013. Noumea.

Statistics Canada (2016) Occupation - National Occupational Classification (NOC) 2016 (693A). Available at: http://www12.statcan.gc.ca/census-recensement/2016/dp-pd/index-eng.cfm [Accessed April 16, 2018].

STECF (2018) *Economic report of the EU fish processing sector 2017*. Publications Office of the European Union, Luxembourg.

STECF (2017) *The 2017 Annual Economic Report on the EU Fishing Fleet (STECF 17-12)*. Publications Office of the European Union, Luxembourg.

Sultana, P., Thompson, P.M. and Ahmed, M. (2002) Women-led fisheries management - A case study from Bangladesh. Penang.

Taniera, T. and Mitchell, J. (1995) Notes from Kiribati. In: *Fishing for Answers: Women and Fisheries in the Pacific Islands*. (ed E. Matthews). Women and Fisheries Network, Suva, pp 29–32.

Teh, L.C.L. and Sumaila, U.R. (2013) Contribution of marine fisheries to worldwide employment. *Fish and Fisheries* **14**, 77–88.

Tetteh, A.S. (2007) Women’s activities in the Ghanaian fishery; The role of social capital. Master’s Thesis. University of Tromsø.

Thalassa (2006) *Fatma, la femme pêcheur du port de Zemmouri*. Daily Motion, Algerie.

Than Thi Hien (2008) Women in Fisheries and Community based Coastal Resource Management in Vietnam: Issues and Challenges. 17.

Thomas, A.S., Mangubhai, S., Vandervord, C., Fox, M. and Thomas, A.S. (2018) Impact of Tropical Cyclone Winston on women mud crab fishers in Fiji. *Climate and Development*, 1–11.

Tietze, U., Lee, R., Siar, S., Moth-Poulsen, T. and Båge, H.E. (2011) Fishing with beach seines. Rome.

Tietze, U., Siar, S., Upare, S.M. and Upare, M.A. (2007) Livelihood and micro-enterprise development opportunities for women in coastal fishing communities in India: Case studies of Orissa and Maharashtra. Rome.

Tiitii, U., Sharp, M. and Ah-Leong, J. (2014) Samoa socioeconomic fisheries survey report 2012/2013. Noumea.

Turner, N. (2003) “Passing on the News”: Women’s Work, Traditional Knowledge and Plant Resource Management in Indigenous Societies of North-western North America. In: *Women & Plants: Gender Relations in Biodiversity Management & Conservation*. (ed P.L. Howard). Zed Books Ltd., London, pp 133–149.

US Bureau of Labor Statistics (2017) Women in the labor force: a databook. Available at: https://www.bls.gov/cps/demographics.htm#women [Accessed April 26, 2018].

US Census Bureau (2016a) American Community Survey: Table B24010, Sex by occupation by employed population 16 years or older. Available at: https://factfinder.census.gov/faces/nav/jsf/pages/index.xhtml [Accessed April 26, 2018].

US Census Bureau (2016b) National Survey of Fishing, Hunting, and Wildlife-Associated Recreation. Available at: https://wsfrprograms.fws.gov/subpages/nationalsurvey/nat_survey2016.pdf [Accessed April 26, 2018].

Valdez-Gardea, G.C. (2001) People’s Response in a Time of Crisis: Marginalization in the Upper Gulf of California. PhD Dissertation. University of Arizona.

Vali, S., Rhodes, K., Au, A., Zylich, K., Harper, S. and Zeller, D. (2014) Reconstruction of Total Fisheries Catches for the Federated States of Micronesia (1950-2010). *Fisheries Centre Working Paper Series*. Vancouver.

Villemur, M. and Angouillant, V.-P. (2015) *Femmes de Mer: 42 Portraits de femmes travaillant dans les secteurs de la pêche et l’aquaculture*. Ministère de l’écologie, du développement durable et de l’énergie, Paris.

Walker, B.L.E. (2002) Engendering Ghana’s Seascape: Fanti Fishtraders and Marine Property in Colonial History. *Society & Natural Resources* **15**, 389–407.

Walker, B.L.E. (2001) Sisterhood and Seine-Nets: Engendering Development and Conservation in Ghana’s Marine Fishery. *Professional Geographer* **53**, 160–177.

Walter, C. (2006) Femmes et Coquillages: Vers une Gestion Participative de la Ressource. Brest.

Wang, Q. and Zhou, Y. (2008) Contributing significantly. *Yemaya* **28**, 5–7.

Westerman, K. and Benbow, S. (2013) The Role of Women in Community-based Small-Scale Fisheries Management: The Case of the South West Madagascar Octopus Fishery. *Western Indian Ocean Journal of Marine Science* **12**, 119–132.

Williams, J. (2006) *Clam Gardens: Aboriginal Mariculture and Canada’s West Coast*. New Star Books, Vancouver.

Williams, J., Rife, A. and Smith, S. (2017) Securing women’s rights and livelihoods. In: *NAAFE Forum 2017: Designing rights-based management systems to achieve social objectives in fisheries*. NAAFE, La Paz.

Williams, S.B. (1996) Economic role of women in fishing communities: a case study of Koko, Nigeria. *Technical Report No. 94*. Cotonou.

Williams, S.B., Hochet-Kibongui, A.-M. and Nauen, C.E. (2005) Gender, fisheries and aquaculture: Social capital and knowledge for the transition towards sustainable use of aquatic ecosystems. *ACP – EU Fisheries Research Report* 16. Brussels.

World Bank (2010) Hidden Harvest: The Global Contribution of Capture Fisheries. Washington, DC.

World Bank (2005) Vietnam Fisheries and Aquaculture Sector Study Final Report. Hanoi.

Xu, S., Xu, Y., Huang, Y. and Zheng, F. (2012) Women’s roles in the construction of new fishing villages in China, as shown from surveys in Zhejiang Province. *Asian Fisheries Science* **25**, 229–236.

Yahaya, J. (2001) Women in small-scale fisheries in Malaysia. In: *International Symposium on Women in Asian*, Vol. 17. (eds M.J. Williams, M.C. Nandeesha, V.P. Orral, E. Tech and C. Poh Sze), Fisheries: Asian Fisheries Society, Chiang Mai, pp 46–48.

Yonger, M. (2002) Approche de la pêcherie récifo-lagonaire de Moorea (Polynésie française): évaluation de la production halieutique et de la population de pêcheurs. Papeete.

Zhao, M., Tyzack, M., Anderson, R. and Onoakpovike, E. (2013) Women as visible and invisible workers in fisheries: A case study of Northern England. *Marine Policy* **37**, 69–76.

Zhao, M., Tyzack, M., Anderson, R. and Onoakpovike, E. (2014) Women in English Fisheries: Roles, Contributions, Barriers and Prospects. In: *Social Issues in Sustainable Fisheries Management*. (eds J. Urquhart, T. Acott, D. Symes and M. Zhao). Springer Netherlands, Dordrecht, pp 233–254.

Zyalya Partal (2018) How the struggle for women’s rights contributes to biodiversity. Available at: http://greenbelarus.info/articles/07-03-2018/kak-borba-za-prava-zhenshchin-sposobstvuet-podderzhaniyu-bioraznoobraziya [Accessed January 12, 2019].
